# A clinically interpretable model for Parkinson’s disease: insights from complementary MRI modalities

**DOI:** 10.1093/braincomms/fcag383

**Published:** 2026-10-13

**Authors:** Qianshi Zheng, Jiaqi Wen, Weijin Yuan, Jianmei Qin, Xiaojie Duanmu, Haoting Wu, Zihao Zhu, Tao Guo, Chenqing Wu, Cheng Zhou, Jingjing Wu, Jingwen Chen, Yuelin Fang, Bingting Zhu, Yaping Yan, Jun Tian, Baorong Zhang, Hongjian He, Minming Zhang, Xiaojun Guan, Xiaojun Xu

**Affiliations:** Department of Radiology, The Second Affiliated Hospital, Zhejiang University School of Medicine, Hangzhou 310009, China; Joint Laboratory of Clinical Radiology, The Second Affiliated Hospital, Zhejiang University School of Medicine, Hangzhou 310009, China; Department of Radiology, The Second Affiliated Hospital, Zhejiang University School of Medicine, Hangzhou 310009, China; Joint Laboratory of Clinical Radiology, The Second Affiliated Hospital, Zhejiang University School of Medicine, Hangzhou 310009, China; Department of Radiology, The Second Affiliated Hospital, Zhejiang University School of Medicine, Hangzhou 310009, China; Joint Laboratory of Clinical Radiology, The Second Affiliated Hospital, Zhejiang University School of Medicine, Hangzhou 310009, China; Department of Radiology, The Second Affiliated Hospital, Zhejiang University School of Medicine, Hangzhou 310009, China; Joint Laboratory of Clinical Radiology, The Second Affiliated Hospital, Zhejiang University School of Medicine, Hangzhou 310009, China; Department of Radiology, The Second Affiliated Hospital, Zhejiang University School of Medicine, Hangzhou 310009, China; Joint Laboratory of Clinical Radiology, The Second Affiliated Hospital, Zhejiang University School of Medicine, Hangzhou 310009, China; Department of Radiology, The Second Affiliated Hospital, Zhejiang University School of Medicine, Hangzhou 310009, China; Joint Laboratory of Clinical Radiology, The Second Affiliated Hospital, Zhejiang University School of Medicine, Hangzhou 310009, China; Department of Radiology, The Second Affiliated Hospital, Zhejiang University School of Medicine, Hangzhou 310009, China; Joint Laboratory of Clinical Radiology, The Second Affiliated Hospital, Zhejiang University School of Medicine, Hangzhou 310009, China; Department of Radiology, The Second Affiliated Hospital, Zhejiang University School of Medicine, Hangzhou 310009, China; Joint Laboratory of Clinical Radiology, The Second Affiliated Hospital, Zhejiang University School of Medicine, Hangzhou 310009, China; Department of Radiology, The Second Affiliated Hospital, Zhejiang University School of Medicine, Hangzhou 310009, China; Joint Laboratory of Clinical Radiology, The Second Affiliated Hospital, Zhejiang University School of Medicine, Hangzhou 310009, China; Department of Radiology, The Second Affiliated Hospital, Zhejiang University School of Medicine, Hangzhou 310009, China; Joint Laboratory of Clinical Radiology, The Second Affiliated Hospital, Zhejiang University School of Medicine, Hangzhou 310009, China; Department of Radiology, The Second Affiliated Hospital, Zhejiang University School of Medicine, Hangzhou 310009, China; Joint Laboratory of Clinical Radiology, The Second Affiliated Hospital, Zhejiang University School of Medicine, Hangzhou 310009, China; Department of Radiology, The Second Affiliated Hospital, Zhejiang University School of Medicine, Hangzhou 310009, China; Joint Laboratory of Clinical Radiology, The Second Affiliated Hospital, Zhejiang University School of Medicine, Hangzhou 310009, China; Department of Neurology, The Second Affiliated Hospital, Zhejiang University School of Medicine, Hangzhou 310009, China; Department of Neurology, The Second Affiliated Hospital, Zhejiang University School of Medicine, Hangzhou 310009, China; Department of Neurology, The Second Affiliated Hospital, Zhejiang University School of Medicine, Hangzhou 310009, China; Department of Neurology, The Second Affiliated Hospital, Zhejiang University School of Medicine, Hangzhou 310009, China; Department of Neurology, The Second Affiliated Hospital, Zhejiang University School of Medicine, Hangzhou 310009, China; Center for Brain Imaging Science and Technology, State Key Laboratory of Brain-Machine Intelligence, Zhejiang University School of Physics, Hangzhou 310027, China; Department of Radiology, The Second Affiliated Hospital, Zhejiang University School of Medicine, Hangzhou 310009, China; Joint Laboratory of Clinical Radiology, The Second Affiliated Hospital, Zhejiang University School of Medicine, Hangzhou 310009, China; Department of Radiology, The Second Affiliated Hospital, Zhejiang University School of Medicine, Hangzhou 310009, China; Joint Laboratory of Clinical Radiology, The Second Affiliated Hospital, Zhejiang University School of Medicine, Hangzhou 310009, China; Department of Radiology, The Second Affiliated Hospital, Zhejiang University School of Medicine, Hangzhou 310009, China; Joint Laboratory of Clinical Radiology, The Second Affiliated Hospital, Zhejiang University School of Medicine, Hangzhou 310009, China

**Keywords:** Parkinson’s disease, clinical decision-support model, quantitative susceptibility mapping, T1-weighted imaging, arterial spin labelling

## Abstract

Accurate identification of Parkinson’s disease may remain challenging in certain clinical scenarios, highlighting the need for a clinical decision-support model based on complementary MRI modalities. The case–control study included 462 participants, split into training [247 Parkinson’s disease, 122 normal controls (NC)] and testing (60 Parkinson’s disease, 33 NC) cohorts to construct and validate a clinical decision-support model. We employed least absolute shrinkage and selection operator and logistic regression analysis to develop the model utilizing quantitative susceptibility mapping, arterial spin labelling and T1-weighted images. The model was assessed for calibration, discrimination and clinical utility, with individual predicted scores correlated to Parkinson’s disease clinical symptoms. The model’s performance was also evaluated independently in multiple system atrophy, progressive supranuclear palsy and essential tremor, with DeLong test comparisons. The model was trained in the training cohort (Parkinson’s disease: 61.39 ± 8.06 years old, 134 males; NC: 61.29 ± 7.56 years old, 54 males) with an area under the ROC curve (AUC) of 0.842 [95% confidence interval (CI) 0.798–0.883] and 0.771 (95% CI 0.657–0.870) in the testing cohort, whose clinical utility was confirmed. The predicted scores were positively correlated with Unified Parkinson’s Disease Rating Scale II (*P* = 0.034) and III (*P* = 0.015), while negatively correlated with the Purdue Pegboard total (*P* = 0.010) and assembly scores (*P* = 0.007). When discriminating each disease group from NC, the model’s performance to identify multiple system atrophy and progressive supranuclear palsy with AUC values of 0.881 and 0.895 was superior than essential tremor with an AUC of 0.709 before false discovery rate correction (multiple system atrophy-NC versus essential tremor-NC: *P* = 0.041; progressive supranuclear palsy-NC versus essential tremor-NC: *P* = 0.010). The model based on complementary MRI modalities was an objective clinical decision-support measure for Parkinson’s disease with potential applications in other parkinsonisms (multiple system atrophy, progressive supranuclear palsy).

## Teaser

Zheng *et al*. developed a clinical decision-support model based on complementary MRI modalities with certain clinical interpretability to effectively identify Parkinson’s disease. The image features constituting the model were applicable to other parkinsonisms (multiple system atrophy, progressive supranuclear palsy), indicating that this model has specificity for parkinsonian disorders.

## Graphical Abstract

**Graphical Abstract:**
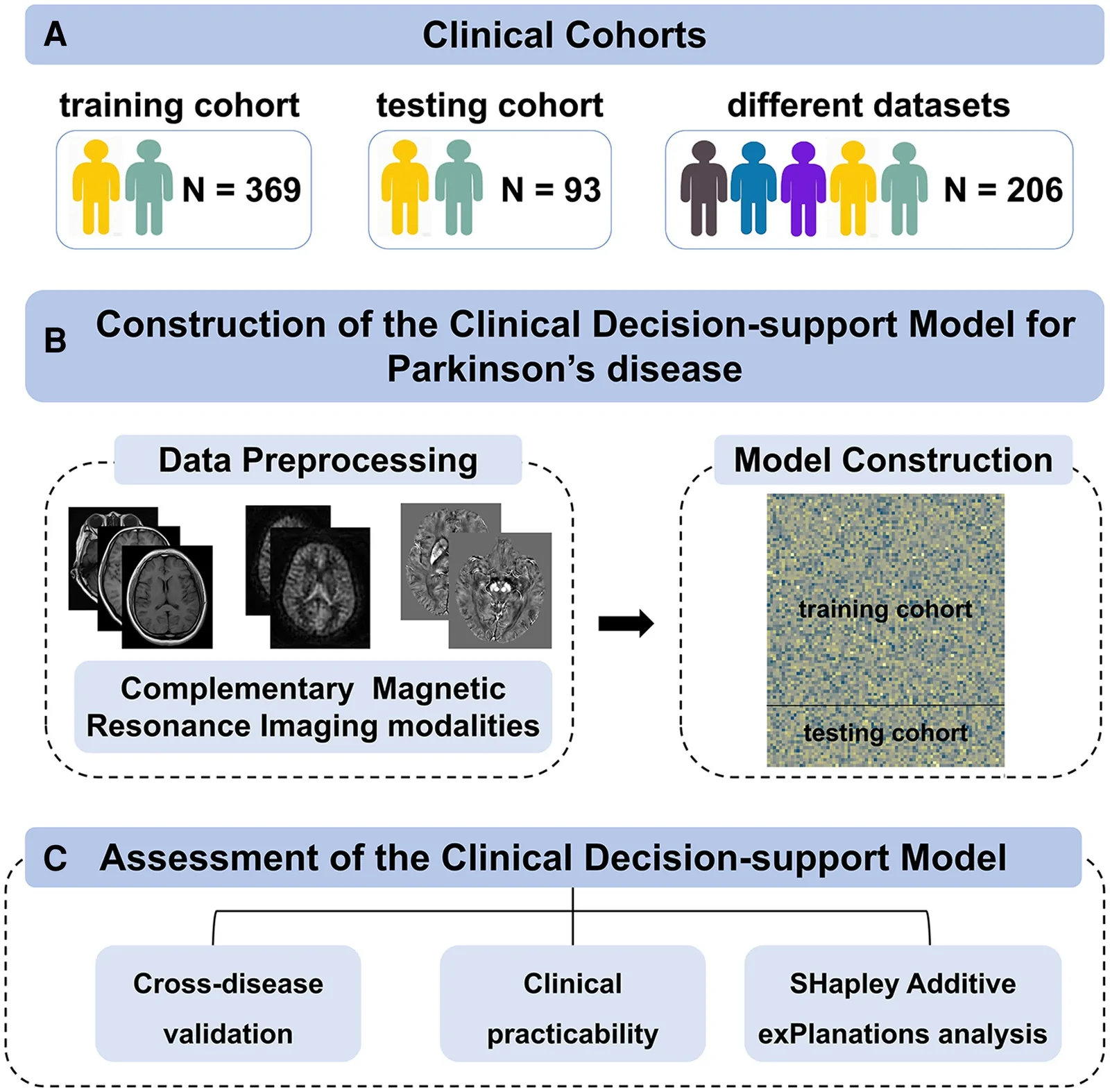

## Introduction

Parkinson’s disease is a common neurodegenerative disease, whose diagnosis currently relies on clinical symptoms.^1^ Recently, the auxiliary diagnostic role of imaging in the modified clinical diagnostic criteria of Parkinson’s disease has received increasing attention,^2^ thus developing objective imaging indicators as an adjunctive imaging support to assist Parkinson’s disease diagnosis and disease assessment is crucial. This could assist in screening and referral of Parkinson’s disease patients to specialized movement disorder centres for further evaluation.

Parkinson’s disease is a multisystem disorder characterized by widespread alterations in brain metabolism, function and structure, which vary considerably across individuals and disease courses.^3^ Non-invasive MRI can *in vivo* assess Parkinson’s disease neurodegeneration in a one-stop scan by comprehensively quantifying brain characteristics, including subcortical iron deposition, functional network abnormalities and cortical degeneration.^3^ Quantitative susceptibility mapping (QSM), an advanced post-processing technology, can effectively distinguish Parkinson’s disease from normal controls (NC) by detecting iron deposition^4^ in the substantia nigra (SN)^5^—a pathological hallmark closely linked to dopaminergic neuron loss in the SN and severity of motor symptoms in Parkinson’s disease.^6^ As a downstream consequence of SN degeneration, the cortico-striatopallido-thalamocortical circuit is typically disrupted, which can directly explain behavioural disability; thus, the functional Parkinson’s disease-related spatial covariance pattern (PDRP) was developed.^7^ Recently, PDRP can be identified using cerebral blood flow measured by non-invasive arterial spin labelling (ASL) technology (ASL_PDRP), which is characterized by hyperactivity in the basal ganglia, pons and cerebellum and hypoactivity in the supplementary motor and parietal cortex, thereby providing diagnostic insights.^8^ ASL_PDRP reveals disease-specific alterations in the cerebral perfusion network, reflecting intrinsic network dysfunction and potentially explaining the impaired motor manifestations observed in Parkinson’s disease.^9^ Furthermore, widespread cortical degeneration, such as frontotemporal and parieto-occipital cortex atrophy, can be spatiotemporally involved in Parkinson’s disease diagnosis.^3,10,11^ T1-weighted structural imaging quantifies alterations in cortical structure, serving as an important imaging indicator of global neurodegenerative changes in Parkinson’s disease.^12^ Despite these efforts to develop imaging markers for Parkinson’s disease, overcoming disease heterogeneity remains the key challenge, as a single indicator and a small sample size are often insufficient. Therefore, by integrating brain metabolic, functional and structural information from large-scale cohorts to capture comprehensive characteristics of Parkinson’s disease degeneration,^13^ it may further promote the development of clinical decision-support models and clinical evaluations.

Moreover, Parkinson’s disease is known as the most common form of parkinsonian disorders, whose prominent feature is parkinsonism.^14^ Another atypical parkinsonian disorder, multiple system atrophy, is an *α*-synuclein disease affecting both the nigrostriatal dopaminergic and cerebellar circuits.^15^ Progressive supranuclear palsy is another common tau-related parkinsonian disorder characterized by widespread cortical and subcortical pathological involvement.^16^ Despite distinct pathologies, these diseases share similar parkinsonian symptoms due to the common impairment of the nigrostriatal system and related circuits.^14^ In contrast, essential tremor is another common movement disorder with symptoms overlapping those of Parkinson’s disease,^17^ but its core pathogenesis arises from the degeneration of the cerebellar-related rather than dopaminergic circuits.^18^ Distinguishing Parkinson’s disease from these disorders or from NC remains clinically challenging, particularly at initial presentation.^19^ Machine learning studies demonstrate that MRI indexes (e.g. grey matter volume or density and subcortical iron deposition) show promising performance in differentiating Parkinson’s disease.^20,21^ However, complex models often involve multiple processing steps, introducing potential bias and complicating clinical translation.^22^ Therefore, we adopted the logistic regression (LR)—a simple yet efficient machine learning method—to develop a clinical decision-support model, thereby simplifying analytical procedures and improving clinical applicability.^23^

So we aimed to integrate measures derived from complementary MRI modalities, including T1-weighted imaging, QSM and pseudo-continuous arterial spin labelling (pCASL), to characterize cortical thickness, subcortical iron deposition and abnormal perfusion patterns in a large Parkinson’s disease cohort, in order to build a clinical decision-support model. The model was further used to evaluate its discriminative efficacy in different datasets (multiple system atrophy, progressive supranuclear palsy and essential tremor), to explore whether the identified image features could also capture the potential shared pathophysiological characteristics between Parkinson’s disease and other parkinsonisms. The study also incorporated various model interpretation methods to test the rationality and provided insights for the clinical assessment of Parkinson’s disease.

## Materials and methods

### Participants

This was a single-centre and retrospective case–control study, which was approved by the Medical Ethics Committee of the Second Affiliated Hospital of Zhejiang University School of Medicine (20170123). All participants provided informed consent for participation and publication of anonymized data, in accordance with the Declaration of Helsinki. Signed forms were held by the investigating team and not shared to protect confidentiality; no identifiable information was included.

Figure 1 showed the research procedures. Cases and controls were recruited independently without individual matching. The subjects with Parkinson’s disease were recruited from clinics of the institute, and NC were recruited from local communities. All participants who met the eligibility criteria during this period were included, resulting in a total of 356 Parkinson’s disease patients and 183 NC originally recruited from August 2014 to December 2023. Diagnosis of Parkinson’s disease was confirmed by a multidisciplinary team composed of neurologists, neurosurgeons and radiologists, according to the UK PD Society Brain Bank^24^ and the Movement Disorder Society clinical diagnostic criteria.^25^ Participants were excluded if they met any of the following conditions: (i) history of cerebrovascular diseases, including stroke and severe microhaemorrhages, (ii) severe cerebral atrophy, (iii) image distortion and artefacts and (iv) other neurological or psychiatric diseases.

**Figure 1:**
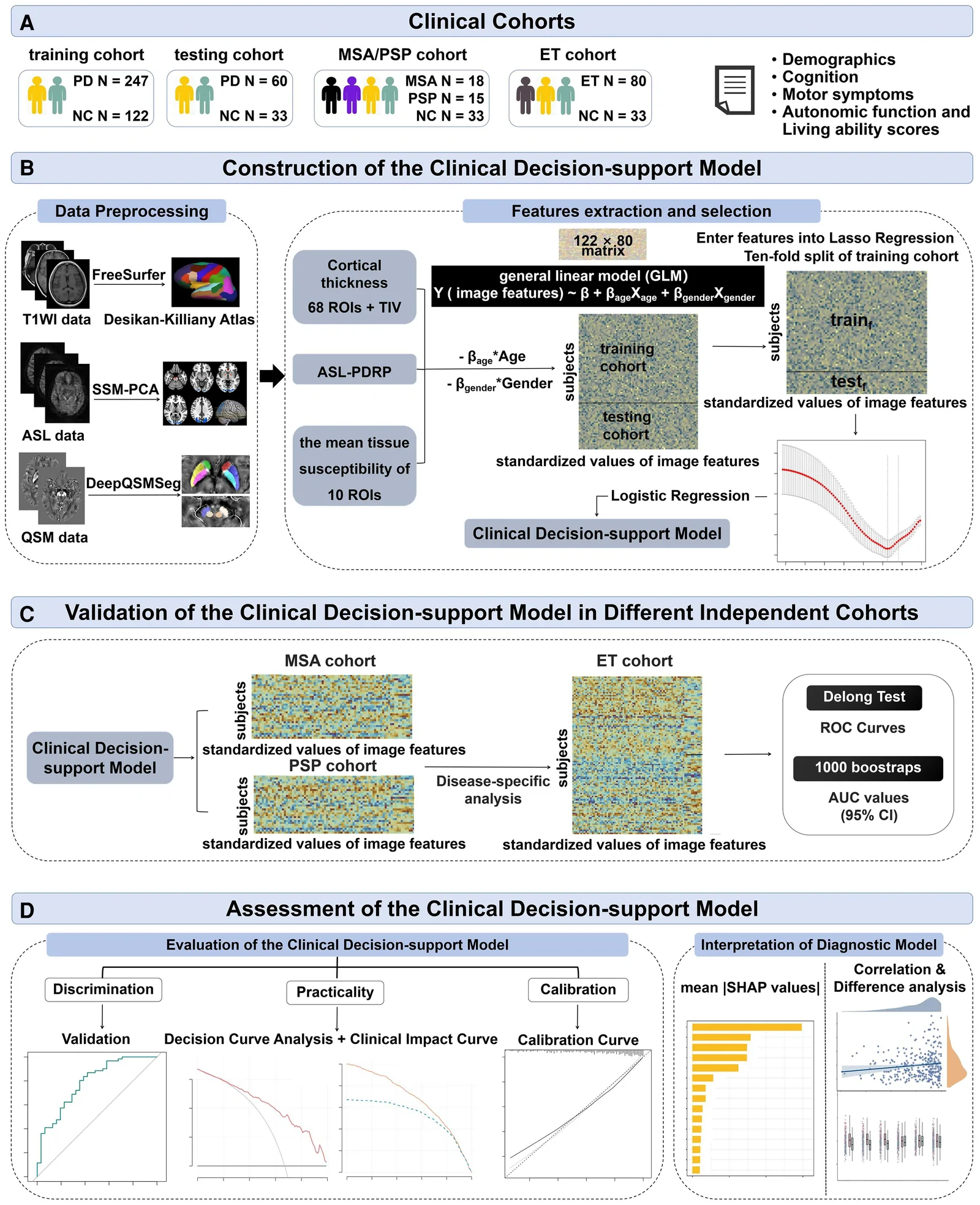
**Overview of the study methodology.** (**A**) The final composition of four clinical cohorts and summary of clinical data collection (PD, *N* = 307; NC, *N* = 155). (**B**) The construction process of the clinical decision-support model. T1-weighted, ASL and QSM images were employed, and a total of 80 image features were extracted. The general linear model was constructed based on 122 NC to control for the influence of age and gender. The optimal number of image features and the specific features of the model were selected by data-driven LASSO regression. The subject-all-feature matrix (80 × 369) and the subject-selected-feature matrix (19 × 369) derived from the training cohort were shown. Then the binary logistic regression analysis was used to construct the model. (**C**) The application and validation of the model to different clinical cohorts. The model was applied to the MSA (*N* = 18), PSP (*N* = 15) and ET (*N* = 80) cohorts. The ROC curves were compared by the DeLong test. The AUC values (95% CI) were obtained by 1000 bootstrapping. (**D**) Assessment of the clinical decision-support model. The discriminative efficiency of the model was repeatedly verified in the testing cohort. We performed the calibration curve, DCA and CIC analysis in both training and testing cohorts. The SHAP analysis was utilized to determine the image features with the highest contribution to the model. Independent-samples *t*-test was used to compare the differences in the normalized values of image features among various groups, and correlation analysis was performed between the individual predicted scores and clinical scale scores. PD, Parkinson’s disease; NC, normal controls; MSA, multiple system atrophy; PSP, progressive supranuclear palsy; ET, essential tremor; T1WI, T1-weighted image; ASL, arterial spin labelling; QSM, quantitative susceptibility mapping; SSM-PCA, scaled sub-profile model and principal component analysis; ROI, region of interest; TIV, total intracranial volume; ASL_PDRP, the PDRP based on ASL; LASSO, least absolute shrinkage and selection operator; train*_f_*, training set of the *f*-th fold; test*_f_* = testing set of the *f*-th fold; ROC, receiver operating characteristic; AUC, area under the ROC curve; CI, confidence interval; SHAP, SHapley Additive exPlanation.

Multiple system atrophy, progressive supranuclear palsy and essential tremor cohorts were recruited to evaluate the discriminative performance of the model in different datasets. Each subject was diagnosed according to clinical criteria: multiple system atrophy and progressive supranuclear palsy were diagnosed by consensus operational criteria,^26,27^ and essential tremor was diagnosed based on the Movement Disorders Society statement.^28^ Finally, 18 multiple system atrophy, 15 progressive supranuclear palsy and 80 essential tremor subjects were included to validate the discriminative efficacy of the model constructed in the training cohort (Fig. 1A). All participants had complete clinical and imaging data. The screening process of all subjects was detailed in Supplementary Fig. 1.

### Clinical scales evaluation

Basic demographic information, including age, gender, disease duration and Mini-Mental State Examination (MMSE) score, was assessed for all patients.^29^ We additionally administered the Purdue Pegboard total and assembly sub-scores, the chair test and the regular walking speed test to quantitatively evaluate Parkinson’s disease patients’ motor symptoms.^30^ The Unified Parkinson’s Disease Rating Scale (UPDRS) I, II and III scores and Hoehn–Yahr (H–Y) stage^31^ were utilized to measure disease conditions of Parkinson’s disease, multiple system atrophy and progressive supranuclear palsy patients. Tremor severity in patients with essential tremor was assessed using the Essential Tremor Rating Scale scores, developed by the Tremor Research Group (www.tremorresearchgroup.org), with the second section of the scale used for rating.^32^

### MRI data acquisition and processing

All participants underwent the same MRI protocol, and all clinical assessments were performed by the same team. Scans were performed on the same 3.0 T MRI scanner (Discovery MR750, GE Healthcare) with an eight-channel head coil. The imaging acquisition equipment and protocols used remained consistent throughout the entire recruitment period. During scanning, participants wore earplugs and had their heads secured with a foam pad. They were instructed to keep their eyes closed and stay awake. If image quality was affected by motion artefacts or other factors, affected sequences were re-scanned to ensure the best quality images for preprocessing. Detailed sequence parameters were described in Supplementary Table 6 and Supplementary Methods A.

T1-weighted images were acquired to measure cortical thickness and total intracranial volume (TIV). All T1-weighted images underwent cortical segmentation using the automatic segmentation software FreeSurfer (version 7.3.2, http://surfer.nmr.mgh.harvard.edu/). Segmentations of 68 cortical regions based on the Desikan–Killiany atlas were inspected individually to ensure acceptable image quality. TIV and bilateral cortical thickness measurements were extracted, yielding a total of 69 image features.

Enhanced susceptibility-weighted angiography was acquired to generate QSM and quantify the iron content of subcortical nuclei. The Susceptibility Tensor Imaging Suite V3.0 software package (https://people.eecs.berkeley.edu/∼chunlei.liu/software.html) was used to calculate tissue susceptibility from phase images. Deep Learning-based End-to-End Tool (DeepQSMSeg)^33^ was utilized to segment 10 subcortical labels, including bilateral SN, RN, caudate nucleus, globus pallidus and putamen in native QSM images. All segmented nuclei images were manually reviewed by a neuroradiologist. Finally, the regional magnetic susceptibility of 10 nuclei from bilateral hemispheres was extracted. Detailed processing procedures were described in Supplementary Methods B.

Perfusion images were acquired using a background-suppressed three-dimensional pCASL sequence with a spiral readout, which is the standard implementation for this sequence on GE platforms. The ASL images were automatically converted into cerebral blood flow maps using Functool software (version 9.4, GE Medical Systems) and preprocessed through Statistical Parametric Mapping (SPM 12). The processing procedures were included in Supplementary Methods C. Preprocessed ASL images were used for principal component analysis and scaled sub-profile model (PCA-SSM), a widely used approach to identify PDRP, implemented in ScAnVP (Scan Analysis and Visualization Processor).^34^ Topographic profile rating (TPR) was subsequently utilized to quantitatively assess the expression of ASL_PDRP at the individual level,^35^ which served as an image feature in testing cohort and other disease cohorts, respectively. Detailed procedures of PCA-SSM and TPR analysis were shown in Supplementary Methods D.

### Normalization and selection of image features

Considering age and gender as potential confounders, we developed a general linear model (GLM) using 122 NC from the training cohort and calculated the resulting effect of age and gender (Fig. 1B). The specific formula was as follows:


$$Y∼\beta +\beta_{\mathrm{age}}X_{\mathrm{age}}+\beta_{\mathrm{gende}r}X_{\mathrm{gender}}$$

Among this formula, *Y* represented brain image features, *X*_age_ denoted the age variable, *X*_gender_ denoted the gender variable, and *β*, *β*_age_ and *β*_gender_ were parameters of GLM. The fitted GLM was then applied to image features of all participants to derive standardized image features with *z*-scores.

The least absolute shrinkage and selection operator (LASSO) regression can minimize potential collinearity among all imaging features by shrinking coefficients of less informative features to zero, thereby achieving feature selection. We used ‘glmnet’ package in R software, with 10-fold cross-validation for internal verification, and measure = ‘deviance’ to identify the optimal parameter (*λ*_min_) in the LASSO model^36^ in the training cohort.

### Construction of the clinical decision-support model

The final cohort was divided into training and testing cohorts according to recruitment time, with a cut-off time of November 2021 and an approximate ratio of 4:1.^37^ Using recruitment time for data splitting helps simulate real-world scenarios.^5^ Binary LR analysis was applied to construct the clinical decision-support model of Parkinson’s disease using image features identified by LASSO regression, and individual predicted scores were obtained. For each independent comparison, the outcome variable was binary (e.g. in the training cohort: Parkinson’s disease versus NC, in the multiple system atrophy-NC cohort: multiple system atrophy versus NC, etc.). The area under receiver operating characteristic (ROC) curve (AUC) values, along with its 95% confidence interval (CI), was estimated using 1000 bootstraps to evaluate the classification performance of the model. In addition, sensitivity, specificity, accuracy, precision and the F2 score were also calculated.

### Evaluation and interpretation of the clinical decision-support model

The goodness of fit, calibration ability and clinical practicability of the model were evaluated in both training and testing cohorts. A calibration curve was used to evaluate how closely the model-predicted probabilities approximated the actual observed outcomes.^38^ The clinical impact curve (CIC), derived from decision curve analysis (DCA), was used to validate clinical applicability of the model and its optimal discriminative value.^39^ Then we applied the SHapley Additive exPlanations (SHAP) method to interpret the model. The higher SHAP values indicate higher probability of being diagnosed with Parkinson’s disease.^40^ Top image features were selected for between-group comparison by using independent-samples *t*-test with false discovery rate (FDR) correction. Pearson correlation analyses were performed between individual predicted scores and UPDRS I–III, chair test, regular walking speed test and Purdue Pegboard sub-scores in Parkinson’s disease patients.

### Exploratory assessment of model discriminative performance across different datasets

We assessed the model’s ability to identify progressive supranuclear palsy, multiple system atrophy and essential tremor patients from NC. The DeLong test with FDR correction was used to compare ROC curves. To explore whether our model might offer an advantage in the early-stage identification of Parkinson’s disease, we applied a strict criterion of ‘symptom onset < 5 years’ to the original independent testing set and finally identified 54 patients with early-stage Parkinson’s disease, forming a new early-stage testing set (PDearly-NC_testing). We then applied our model based on complementary MRI modalities directly to this new cohort to evaluate its performance.

### Robustness analysis: evaluating the impact of class imbalance

To examine whether class imbalance in the training cohort introduced bias into the model, we performed a robustness analysis using the synthetic minority over-sampling technique (SMOTE). SMOTE analysis was applied exclusively to the training cohort to avoid data leakage, synthetically oversampling the minority class (NC) from 122 to 247 to achieve a fully balanced training set (Parkinson’s disease:NC = 247:247). The original, imbalanced testing cohort was kept untouched for final model evaluation. In this independent testing cohort, the performance metrics were calculated for both the original model and the SMOTE-balanced model. The ROC curves of the two models on the testing cohort were compared using the DeLong test.

### Other statistical analysis

Statistical analyses and visualization were performed using SPSS 25.0 and R software version 4.2.1. The Kolmogorov-Smirnov test was applied to test the normal distribution of data. The independent-samples *t*-test was used for inter-group comparison of normally distributed data, while the Mann–Whitney U-test was used for non-normally distributed data. The χ² test evaluated gender and H–Y stage distribution differences. All tests were two-tailed with significance set at *P* < 0.05. No subgroup or interaction analyses were conducted, as they were beyond the scope of this clinical decision-support model development study. The authors confirm that the data codes supporting the findings of this study are available online.

## Results

### Demographic and clinical information

As depicted in Table 1, a total of 462 participants were divided into training (247 Parkinson’s disease, 122 NC) and testing (60 Parkinson’s disease, 33 NC) cohorts for model construction and validation. Furthermore, 49 Parkinson’s disease and 28 NC were excluded due to the aforementioned exclusion criteria (Supplementary Fig. 1). Gender distribution differed significantly in the testing cohort (*P* = 0.045). NC showed higher MMSE scores than Parkinson’s disease in both cohorts (training cohorts, *P* < 0.001; testing cohorts, *P* = 0.005). Multiple system atrophy and progressive supranuclear palsy cohorts showed lower MMSE scores compared to NC (multiple system atrophy-NC, *P* = 0.001; progressive supranuclear palsy-NC, *P* < 0.001). Furthermore, progressive supranuclear palsy patients were slightly older (progressive supranuclear palsy-NC, *P* = 0.029) (Supplementary Table 1). No significant difference in age (*P* = 0.440) and MMSE (*P* = 0.170) was found between essential tremor and NC groups (Supplementary Table 2).

**Table 1:** Demographic and clinical information of the recruited subjects of the training cohort and testing cohort

	Training cohort		Testing cohort		*P*_1_	*P*_2_
	PD	NC	PD	NC		
	247	122	60	33		
Age (yr)	61.39 ± 8.06	61.29 ± 7.56	61.06 ± 7.51	60.62 ± 6.48	0.865	0.758
Gender (F/M）	113:134	68:54	27:33	22:11	0.079	**0.045**
MMSE	27.00 (4.00)	29.00 (3.00)	26 (6.00)	29.00 (4.00)	***P* < 0.001**	**0.005**
Disease duration (yr)	3.01 (4.28)		2.06 (2.78)			
UPDRS I score	1.00 (2.00)		1.00 (2.00)			
UPDRS II score	8.00 (6.00)		8.00 (6.00)			
UPDRS III score	21.00 (18.00)		24.00 (18.00)			
Hoehn–Yahr stage	2.50 (2.00–2.50)		2.00 (2.00–2.50)			
Purdue Pegboard total scores	31.64 ± 8.40		32.63 ± 7.39			
Purdue Pegboard assembly scores	17.44 ± 6.04		17.33 (7.67)			

The one-sample Kolmogorov–Smirnov test was applied to test the normal distribution of data. Normally distributed data was presented as the mean ± SD, while non-normally distributed data was shown as the median (interquartile range). The median (first quartile, third quartile) was shown for the Hoehn–Yahr stage. The independent-samples *t*-test was used for each inter-group comparison of normally distributed data, while the Mann–Whitney U-test was used for non-normally distributed data. *P* < 0.050 was considered statistically significant and indicated in bold.

PD, Parkinson’s disease; NC, normal controls; MMSE, the Mini-Mental State Examination; UPDRS, United Parkinson’s Disease Rating Scale. *P*_1_, comparisons between PD and NC groups in the training cohort; *P*_2_, comparisons between PD and NC groups in the testing cohort.

### The clinical decision-support model construction and assessment

Age and gender were included as confounders in the adjusted analyses because they are established risk factors for Parkinson’s disease and are known to affect brain imaging metrics. Internal 10-fold cross-validation of LASSO regression determined the optimal feature number at *λ*_min_ as 19 (Fig. 2A and B). These image features consisted of 14 cortical structure features (lh_caudalmiddlefrontal, lh_inferiorparietal, lh_lingual, lh_medialorbitofrontal, lh_pericalcarine, lh_rostralmiddlefrontal, rh_caudalanteriorcingulate, rh_fusiform, rh_inferiortemporal, TIV, rh_isthmuscingulate, rh_parsorbitalis, rh_rostralanteriorcingulate, rh_supramarginal), four QSM features (R_caudate, R_pallidum, L_SN, R_SN) and ASL_PDRP. These image features were retained to develop the clinical decision-support model of Parkinson’s disease by using binary LR analysis, and the regression coefficients of the model served as weights for each image feature to calculate the individual predicted score (Supplementary Table 5).^41^

**Figure 2:**
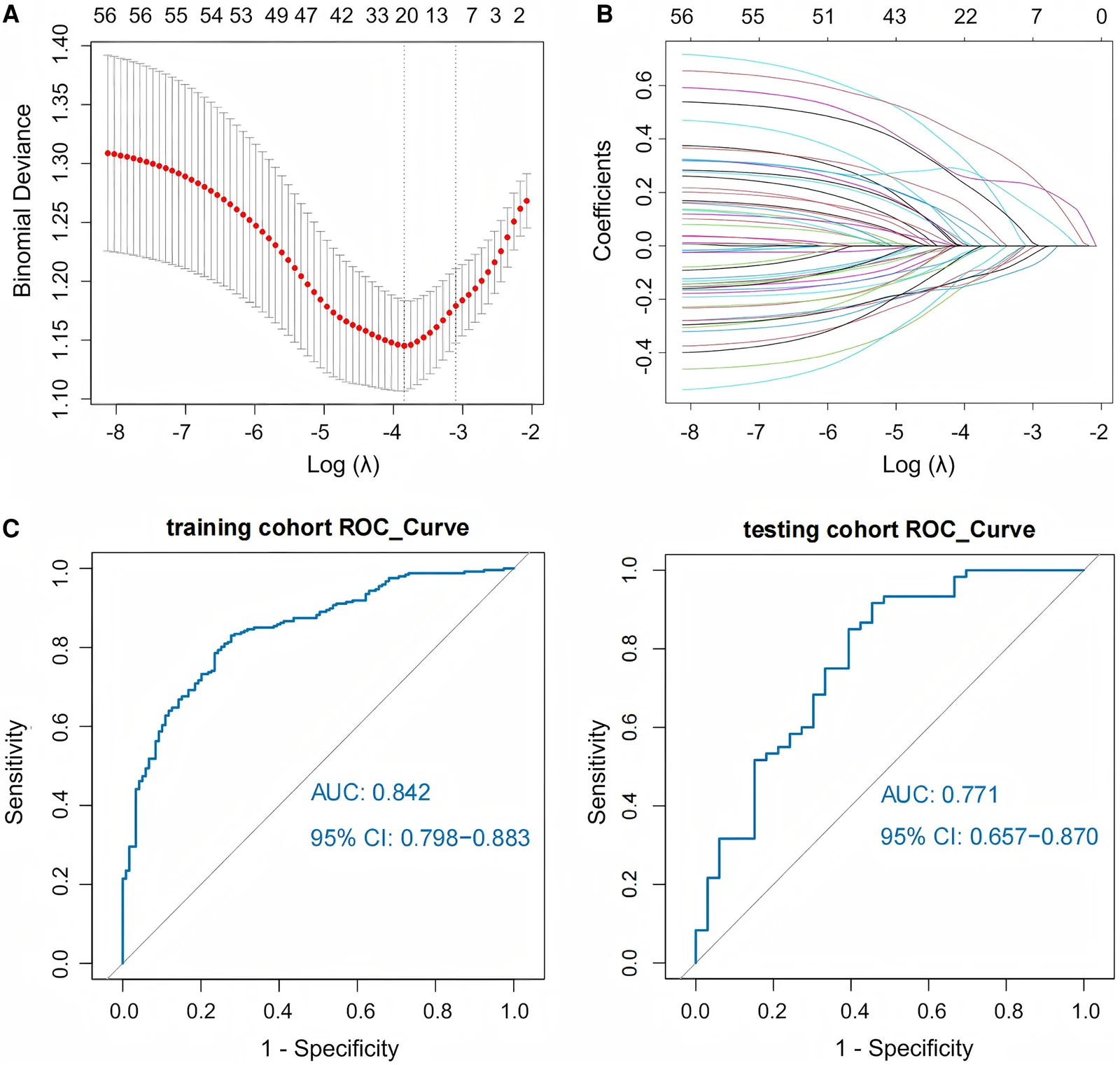
**The results of LASSO regression and ROC curve analysis of the clinical decision-support model in the training and testing cohorts.** (**A**) Log lambda (*λ*) and the error model from LASSO regression by using 10-fold cross-validation. The two dashed vertical lines revealed *λ*_min_ (left, the value of *λ* when the error model was minimal) and *λ*.1se (right, the error model within a standard error range of *λ*), respectively. We selected *λ*_min_ as the optimal values for confirming the number of image features. (**B**) Log (*λ*) and the LASSO regression coefficient. Identify the optimal *λ* or penalty coefficient from LASSO regression with 10-fold cross-validation. (**C**) The AUC with 95% CI was calculated by 1000 bootstrapping in the training cohort (PD, *N* = 247; NC, *N* = 122) and the testing cohort (PD, *N* = 60; NC, *N* = 33). PD, Parkinson’s disease; NC, normal controls; LASSO, least absolute shrinkage and selection operator; AUC, area under the ROC curve; CI, confidence interval.

In the training cohort, the model distinguished Parkinson’s disease patients from NC with an AUC of 0.842 (95% CI 0.798–0.883), accompanied by a specificity of 0.723, a sensitivity of 0.830, an accuracy of 0.762, a precision of 0.794 and an F2 score of 0.857. Independent validation showed an AUC of 0.771 (95% CI 0.657–0.870) with a specificity of 0.546, a sensitivity of 0.917, an accuracy of 0.634, a precision of 0.796 and an F2 score of 0.616 (Fig. 2C; Table 2). The Hosmer-Lemeshow test was used to assess goodness of fit, with *P* value greater than 0.05 indicating a good fit (*P* = 0.395).^42^ The calibration curve of the model in the training and testing cohorts both presented good agreement with the actual observed outcomes [mean absolute error (MAE): training cohort = 0.034; testing cohort = 0.079] (Fig. 3A and B). DCA assesses the utility of the model for decision-making by plotting net benefit (NB) within a clinically reasonable risk threshold.^43^ DCA demonstrated that within a threshold probability of ∼0.7–0.8, the model could achieve greater NB in Parkinson’s disease identification (Fig. 3C and D), highlighting its implications for improving discriminative accuracy in clinical decision-making. CIC suggested that the number of subjects predicted to be at high risk closely matched the number of true Parkinson’s disease in both cohorts, with a lower probability threshold in the training cohort (Fig. 3E and F). These findings indicated that the model can accurately identify high-risk Parkinson’s disease patients and assist in screening under different risk judgment criteria.

**Figure 3:**
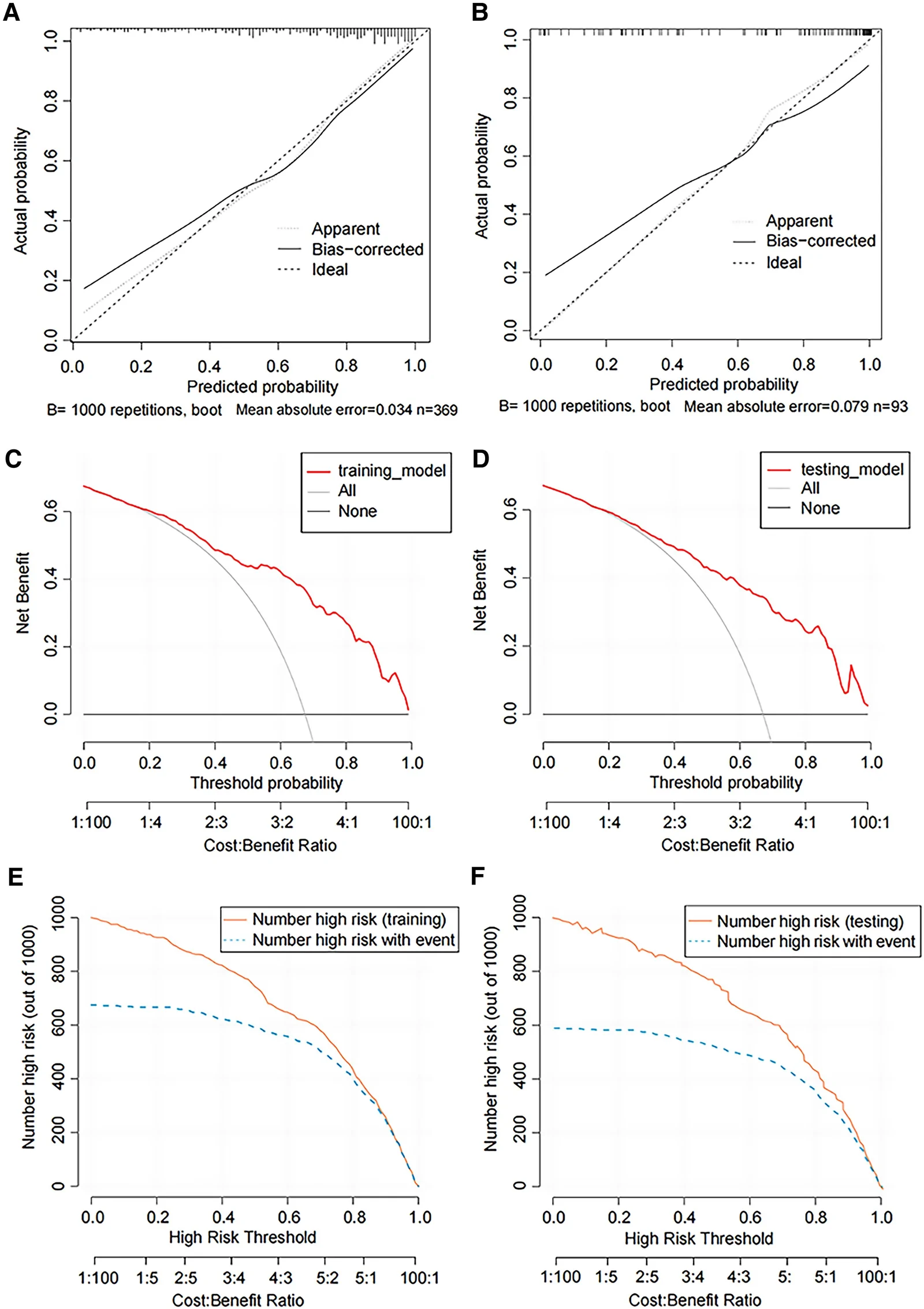
**Assessment the clinical decision-support model.** The calibration curve analysis of the clinical decision-support model in the training cohort [(**A**) PD, *N* = 247; NC, *N* = 122; MAE = 0.034] and the testing cohort [(**B**) PD, *N* = 60; NC, *N* = 33; MAE = 0.079]. The dashed line represented the perfect prediction of the model. The dotted line represented the apparent estimates of predicted versus observed values; meanwhile, the solid line (on behalf of bias) represented the corrected estimates by employing 1000 bootstrap samples. The DCA curve analysis of the model in the training cohort (**C**) and the testing cohort (**D**). The results of the DCA curve of the model depicted that a higher net benefit for PD prediction by using the model compared to intervening either no or all patients. The model was represented by the solid line on the right. The horizontal solid line indicated that if all subjects were negative samples without any intervention, the net benefit was zero. The solid line on the left revealed that all subjects were positive samples, and the intervention was performed on each one. The net benefit was calculated by subtracting the proportion of patients with false positives from the proportion of true positives. The CIC analysis of the model in the training cohort (**E**) and the testing cohort (**F**). In the CIC of the model, the solid curve represented the number of subjects at high risk who were classified by the model as positive (PD) at each threshold probability. The dotted curve represented the number of true positives (PD) at each threshold probability. PD, Parkinson’s disease; DCA, decision curve analysis; CIC, clinical impact curve; MAE, mean absolute error.

**Table 2:** Discrimination performance index in different independent cohorts

Model	Specificity	Sensitivity	Accuracy	Precision	F2 score	AUC values (95% CI)
PD-NC_training	0.723	0.830	0.762	0.794	0.857	0.842 (0.798–0.883)
PD-NC_testing	0.546	0.917	0.634	0.796	0.616	0.771 (0.657–0.870)
MSA-NC	0.849	0.833	0.784	0.640	0.825	0.881 (0.768–0.993)
PSP-NC	0.697	0.999	0.792	0.609	0.843	0.895 (0.808–0.982)
ET-NC	0.697	0.663	0.700	0.795	0.779	0.709 (0.600–0.819)
PDearly-NC_testing	0.606	0.870	0.644	0.781	0.623	0.773 (0.668–0.878)

The calculation of the area under the ROC curve (AUC) with the 95% confidence interval (CI) was calculated by 1000 bootstrapping. Specificity, sensitivity, accuracy, precision and F2 score corresponding to the optimal cut-off value were performed on the model to assess the ability for PD identification in all cohorts. The optimal cut-off value of the training cohort was 0.587; the optimal cut-off value of the testing cohort was 0.184; the optimal cut-off values of MSA-NC, PSP-NC and ET-NC cohorts were 0.664, 0.380 and 0.594, respectively; the optimal cut-off value of the PDearly-NC_testing cohort was 0.243. The F2 score is a weighted harmonic mean that places greater emphasis on sensitivity, and it is calculated as 5 × (precision × sensitivity)/(4 × precision + sensitivity).

PD, Parkinson’s disease; PDearly, early PD samples (54 PD) from the testing cohort; NC, normal controls; MSA, multiple system atrophy; PSP, progressive supranuclear palsy; ET, essential tremor.

### Correlation analysis and image features selection

The final LR model can be expressed as a linear risk score (the individual predicted score). The full model equation and corresponding regression coefficients are provided in Supplementary Table 5.

The individual predicted scores of Parkinson’s disease patients were positively correlated with UPDRS II (*R* = 0.121; *P* = 0.034) and III (*R* = 0.138; *P* = 0.015) scores (Fig. 4B and C) and negatively with Purdue Pegboard total (*R* = −0.209; *P* = 0.010) and assembly scores (*R* = −0.220; *P* = 0.007) (Fig. 4D and E). No significant correlation was observed between predicted scores and UPDRS I (*R* = −0.097; *P* = 0.089) (Fig. 4A). The detailed results were provided in Supplementary Table 11.

**Figure 4:**
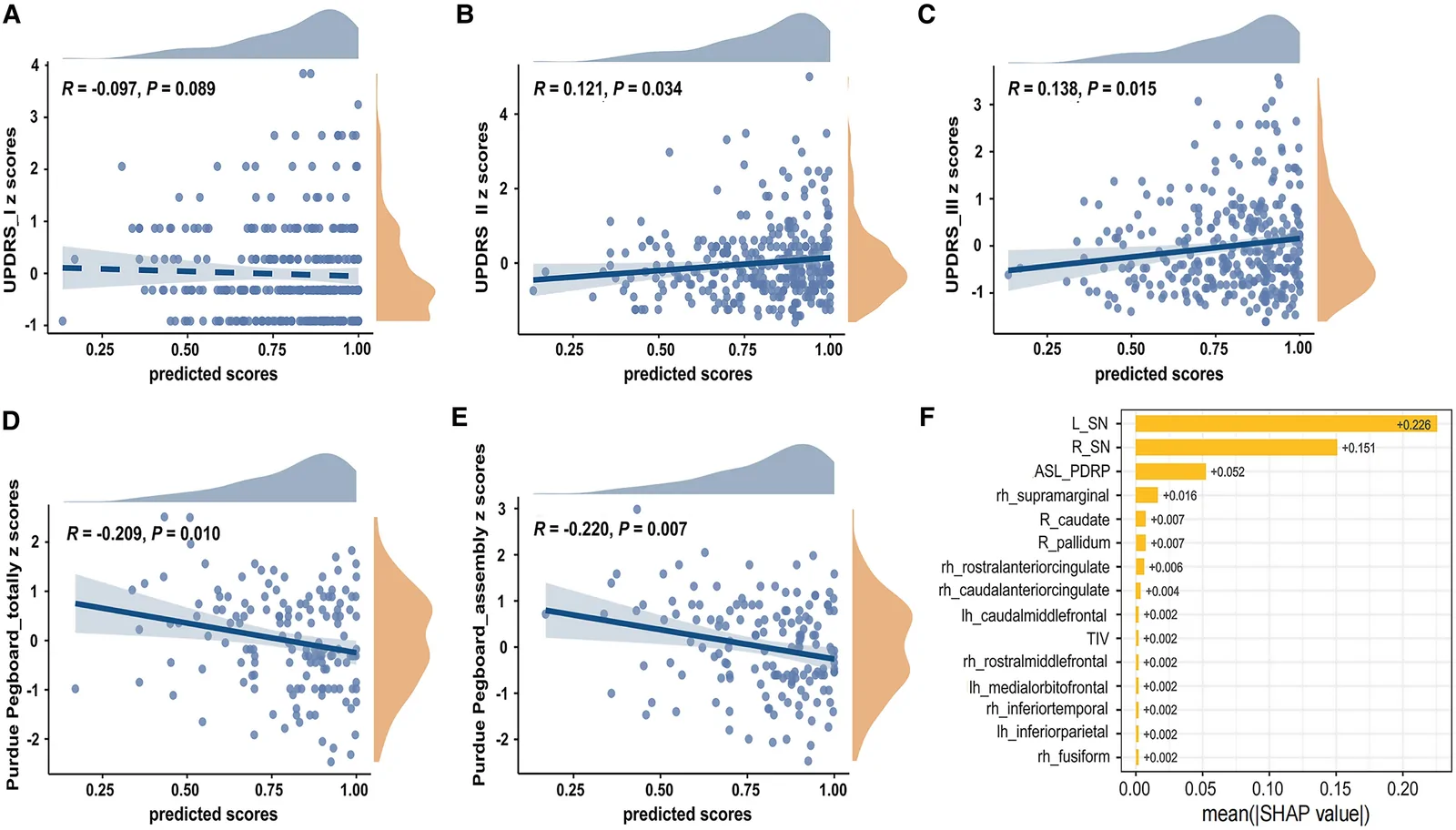
**Interpretation of the clinical decision-support model.** (**A–E**) The Pearson correlation analysis of UPDRS I–III *z-*scores and the Purdue Pegboard total and assembly scores with the individual predicted scores in the included PD patients (training cohort + testing cohort, *N* = 307). Each data point represents an individual participant. The individual predicted scores were positively correlated with UPDRS II (*R* = 0.121; *P* = 0.034) and III (*R* = 0.138; *P* = 0.015) scores and negatively with the Purdue Pegboard total (*R* = −0.209; *P* = 0.010) and assembly scores (*R* = −0.220; *P* = 0.007). (**F**) SHAP summary bar plot for the top 10 image features contributing to the clinical decision-support model. Ranking of variable importance based on the mean I SHAP value I. The plot ranked variable importance based on the mean I SHAP value I, which represented the average impact of each feature on the model’s predictions. The higher the value, the greater the influence of that feature on the outcome, which meant the higher probability of being identified with PD. PD, Parkinson’s disease; SHAP, SHapley Additive exPlanation; SN, substantia nigra; TIV, total intracranial volume. UPDRS, Unified Parkinson’s Disease Rating Scale; ASL_PDRP, the PDRP based on ASL; LH, left hemisphere; RH, right hemisphere.

The SHAP method selected the top six image features contributing to model prediction, including L_SN, R_SN, ASL_PDRP, rh_supramarginal, R_caudate and R_pallidum (Fig. 4F). The group comparison for all image features comprising the model across all cohorts was presented in Supplementary Tables 7–10. ASL_PDRP and iron deposition in the bilateral SN in the training and testing cohorts were all higher in Parkinson’s disease than in NC (Supplementary Figs. 2 and 3; training cohort: ASL_PDRP, *P* < 0.001; R_SN, *P* < 0.001; L_SN, *P* < 0.001; testing cohort: ASL_PDRP, *P* = 0.015; R_SN, *P* = 0.008; L_SN, *P* = 0.008.). Similar findings were observed in progressive supranuclear palsy patients compared to NC (Supplementary Fig. 4A–C; progressive supranuclear palsy-NC: ASL_PDRP, *P* < 0.001; R_SN, *P* < 0.001; L_SN, *P* < 0.001). ASL_PDRP was also higher in multiple system atrophy than in NC (Supplementary Fig. 5A; multiple system atrophy-NC: *P* = 0.007), while lower expression in essential tremor compared to Parkinson’s disease (Supplementary Fig. 6A; ET-PD: *P* = 0.017). Iron deposition in the left SN in multiple system atrophy were higher than NC (Supplementary Fig. 5C; multiple system atrophy-NC: *P* = 0.007). Besides, iron deposition in the right pallium and caudate was higher in progressive supranuclear palsy (Supplementary Fig. 4D and E; progressive supranuclear palsy-NC: R_pallium, *P* = 0.001; R_caudate, *P* = 0.030; progressive supranuclear palsy-PD: R_pallium, *P* < 0.001; R_caudate, *P* = 0.018). Right supramarginal gyri’s thickness was thinner in progressive supranuclear palsy (Supplementary Fig. 4F; progressive supranuclear palsy-NC: *P* = 0.017). These image features showed no significant differences in multiple system atrophy-Parkinson’s disease and essential tremor-NC groups (Supplementary Figs. 5 and 6).

### The model’s discrimination efficiency in different independent cohorts

The model was then applied to the testing set, different disease sets and the early-stage testing set. The model performed well in distinguishing multiple system atrophy and progressive supranuclear palsy patients from NC, with an AUC of 0.881 (95% CI 0.768–0.993) in multiple system atrophy (multiple system atrophy-NC), 0.895 (95% CI 0.808–0.982) in progressive supranuclear palsy (progressive supranuclear palsy-NC) and 0.709 (95% CI 0.600–0.819) in essential tremor (essential tremor-NC). The AUC values obtained in the multiple system atrophy-NC and progressive supranuclear palsy-NC cohorts were higher than the essential tremor-NC cohort (multiple system atrophy-NC versus essential tremor-NC: *P* = 0.041; progressive supranuclear palsy-NC versus essential tremor-NC: *P* = 0.010) before FDR correction (Supplementary Table 3). We further assessed the model’s ability to distinguish early-stage Parkinson’s disease from NC. The results showed an AUC of 0.773 (95% CI 0.668–0.878) with a specificity of 0.606, a sensitivity of 0.870, an accuracy of 0.644, a precision of 0.781 and an F2 score of 0.623, as Table 2 depicted.

### Robustness analysis to evaluate the impact of class imbalance

The SMOTE analysis yielded the following results: the DeLong test revealed no significant difference in ROC curves between the original model (AUC = 0.771, 95% CI 0.657–0.870) and the SMOTE-balanced model (AUC = 0.777, 95% CI 0.677–0.878) on the testing cohort (*P* = 0.324). Detailed results were presented in Supplementary Table 4.

## Discussion

This study utilized complementary MRI modalities to develop a clinical decision-support model for Parkinson’s disease. Key findings included the following: (i) The model based on complementary MRI modalities achieved AUC values of 0.842 and 0.771 in the training and testing cohorts, respectively. Individual predicted scores for Parkinson’s disease showed significant correlations with UPDRS II and III and Purdue Pegboard scores. (ii) The discriminative performance of the model varied across other diseases cohorts: when discriminating disease groups from NC, it excelled in distinguishing multiple system atrophy and progressive supranuclear palsy patients with AUC values of 0.881 and 0.895, respectively, but performed less effectively in the essential tremor cohort with an AUC of 0.709. The model we developed leveraged the growing wealth of MRI data from cerebral perfusion, metabolism and structure research. The results and validation suggest that the model holds potential for clinical utility.

This study constructed a clinical decision-support model based on a large Parkinson’s disease population and validated it in an independent Parkinson’s disease population by employing information of brain morphometry, iron and perfusion patterns. Previous MRI-based studies reported their findings with various discriminative efficiencies of Parkinson’s disease ranging from 71 to 92%.^44,45^ Several studies tried to employ complementary MRI data to cover more cerebral degeneration of Parkinson’s disease.^13,46^ Multimodal and multi-sequence models can further improve the classification performance of Parkinson’s disease, though with a testing accuracy of only 70% as reported.^47^ Generally, the discriminative performance of the model varies depending on disease heterogeneity,^48^ highlighting the need for larger sample sizes and prevention of overfitting. Our model benefitted from a larger sample size, independent validation and the integration of complementary imaging modalities, contributing to its improved performance. Compared with basic sequences used in clinical practice, our model is not intended to replace expert radiological assessment, but may serve as a reproducible, quantitative and relatively objective adjunctive tool, particularly in situations where imaging findings are subtle, interpretation may vary across readers or specialist experience is limited. The correlation between individual predicted scores and clinical symptoms suggested that this objective indicator might help assess the disease condition. However, the low correlation coefficients, though statistically significant, suggest limited clinical interpretability. This likely arises from the marked heterogeneity of Parkinson’s disease, which is manifested not only in clinical presentation but also in potential neuroimaging features among individuals. Such heterogeneity may attenuate univariate correlations. The discrimination, calibration and clinical practicality of our model further indicated the relatively consistent performance across cohorts, suggesting that the model might possess practical clinical values. In clinical practice, the selection of the continuous risk threshold of DCA should be guided by the clinical goal.^49^ The DCA results indicated that within this threshold range, the model provides a higher NB than the treat-all strategy, making it suitable for scenarios in Parkinson’s disease identification. However, it should be noted that different threshold settings correspond to different clinical needs and that predictive values are related to disease prevalence. Therefore, when applying the model in clinical practice, the interpretation of its predictive values should be contextualized accordingly. Due to the heterogeneity of Parkinson’s disease and the limitations of the sample size, the above threshold range still requires independent external validation before it can be recommended for clinical use.

To preliminarily explore the model’s potential in broader clinical situations, we performed exploratory analyses to test the model’s ability to distinguish patients with multiple system atrophy, progressive supranuclear palsy and essential tremor from NC. The results suggested that although progressive supranuclear palsy, multiple system atrophy and Parkinson’s disease are driven by different molecular pathologies, early pathological changes may disrupt some overlapping subcortical motor networks, particularly the nigrostriatal and the basal ganglia–thalamus–cortex circuits.^14,50,51^ Such convergence at the network level may contribute to the similar clinical manifestations seen across these disorders. The value of a complementary approach lies in its ability to capture distributed alterations within these overlapping networks, rather than relying on abnormalities in any single region. Multiple system atrophy and progressive supranuclear palsy patients often exhibited more severe clinical symptoms and extensive pathological degeneration, particularly in progressive supranuclear palsy,^16^ which might explain the effectiveness of the model with a relatively higher performance in these two cohorts (multiple system atrophy/progressive supranuclear palsy versus NC). Essential tremor is a neurodegenerative disease with benign tremor symptom,^17^ characterized by cerebellar degeneration with the dopaminergic circuit preserved.^18^ Thus, essential tremor served as an ideal negative disease control, and the model’s poor performance confirmed its specificity for parkinsonian disorders. These findings suggested that though the model was constructed from Parkinson’s disease patients, other parkinsonian disorders might also benefit from it. We also performed exploratory analyses to test the model’s ability to distinguish early-stage Parkinson’s disease patients from NC. The results indicated that the model’s overall discriminative performance in the early-stage testing set was comparable to that in the original testing set. These findings suggest that our model may identify imaging features associated with Parkinson’s disease that are relatively independent of disease duration, supporting its potential applicability across different disease stages.

We further applied SHAP analysis to ultimately identify key image features with high contributions to the model.^4^ Regarding iron deposition in the basal ganglia region, Parkinson’s disease patients showed higher bilateral SN iron deposition than NC. Increased SN iron deposition reflects the core pathology of Parkinson’s disease, where excessive iron accumulation causes oxidative stress and neurodegeneration in this motor-regulating region.^4^ Further analysis revealed that iron deposition in the right caudate and globus pallidus was more pronounced in progressive supranuclear palsy patients, which is consistent with the more extensive cortical–subcortical pathological involvement and severe motor symptoms observed in progressive supranuclear palsy.^16^ In terms of cerebral perfusion, ASL_PDRP was another core feature of the model. Parkinson’s disease patients showed significantly elevated ASL_PDRP than the NC group, indicating abnormal activation within motor circuits,^9^ consistent with the pathophysiological changes of Parkinson’s disease. This indicator is also closely related to the motor symptoms of Parkinson’s disease.^52^ Notably, multiple system atrophy and progressive supranuclear palsy patients showed a trend towards higher ASL_PDRP. These findings suggested that Parkinson’s disease, multiple system atrophy and progressive supranuclear palsy may share involvement of certain core neural circuits (e.g. the cortico-basal ganglia-thalamocortical loop) in their pathological mechanisms. This shared circuit-level pathophysiology may underlie the overlapping clinical features of motor impairment observed across these disorders.^53^ Regarding the image feature of cortical thickness, cortical degeneration appeared widespread in Parkinson’s disease, as 14 out of 19 image features were cortical thickness measures for model construction. These involved cortical regions that extensively regulate emotion, cognition and motor function, with thinning reflecting neuronal loss underlying Parkinson’s disease dysfunction.^54^ Inter-group comparisons revealed differences in the degree of cortical thickness impairment among diverse diseases, which could lead to divergent clinical symptoms.^55,56^ Both progressive supranuclear palsy and multiple system atrophy showed greater right supramarginal gyrus thinning, which might explain the more severe symptoms of cognitive decline and emotional regulation disorder in these patients.^54,57^ These structural metrics may offer useful information for disease identification and assessment. Therefore, the integration of the above imaging features provided a more comprehensive clinical interpretation of the model.

Several limitations of this study should be acknowledged. Firstly, data originated from a single centre using a single 3 T MRI scanner, which limits the generalizability of our model to other clinical settings. In MRI-based studies, cross-site scanning heterogeneity—stemming from differences in scanners, acquisition protocols and post-processing pipelines—represents a major challenge for model robustness.^58-60^ To address these challenges, future studies should incorporate multicentre cohorts with standardized acquisition protocols and consistent post-processing pipelines. We will also employ data harmonization methods, such as neuroCombat or advanced unsupervised algorithms, to systematically reduce batch effects and scanner-related variability, thereby enhancing the model’s stability in multicentre settings.^61,62^ Secondly, the limited number of multiple system atrophy and progressive supranuclear palsy patients in our study is due to the rarity of these diseases. It merely enabled an initial analysis in the current study. Hopefully, we can build and validate specific models for them with continuous recruitment. The proposed future directions for refinement include expanding the sample size, optimizing feature selection, developing dedicated clinical decision-support models (e.g. different clinical subtypes) and systematically comparing our model with established imaging markers (e.g. P/M ratio, MRPI) in independent cohorts. The ultimate goal is to achieve more precise differentiation between Parkinson’s disease and other parkinsonisms. Thirdly, this was a retrospective study that might cause bias from historical data. Larger prospective and blinded validation studies with longer follow-up should be considered to offer stronger clinical evidence and to better evaluate the model’s clinical value in discriminative trajectory prediction in the future. Finally, the model is currently more suitable for semi-automated analysis in institutions equipped with a dedicated imaging post-processing platform, rather than on a standard MRI scanner console. Future studies should further optimize the current imaging-processing pipeline, improve automation, enhance integration with clinical workflows and explore the feasibility of deployment on hospital-side servers.

## Conclusion

In conclusion, the model based on complementary MRI modalities demonstrated good discriminative performance and may represent a promising objective clinical decision-support tool for the identification of Parkinson’s disease, including in the early stages of the disease, with favourable clinical interpretability. The model’s promising performance in distinguishing other parkinsonisms from NC may offer insights into the shared neurobiological mechanisms of parkinsonian disorders, contributing to a better understanding of Parkinson’s disease pathophysiology.

## Supplementary Material

fcag383_Supplementary_Data

## Data Availability

All data generated or analysed during this study are included in this published article. The main analysis codes generated for this work are available in the GitHub repository (https://github.com/Xlab-Xu/Construction-of-the-PD-diagnostic-model/tree/master). Clinical and neuroimaging data can be shared on reasonable request by contacting the corresponding authors (xxjmailbox@zju.edu.cn, xiaojunguan1102@zju.edu.cn).
