# Supplementary material for: A clinically interpretable model for Parkinson’s disease: insights from complementary MRI modalities
Source: Brain Commun. 2026 Oct 13;8(5):fcag383. doi: 10.1093/braincomms/fcag383 (PMC13684402; doi:10.1093/braincomms/fcag383)
Supplement: Supplementary file 1 [file fcag383_supplementary_data.docx]

**A clinically interpretable model for Parkinson’s disease: insights from complementary MRI modalities**

**Supplementary appendix**

SUPPLEMENTARY TABLES	4

Supplementary Table 1. Demographic and clinical information of the MSA and the PSP cohort	4

Supplementary Table 2. Demographic and clinical information of the ET cohort.	5

Supplementary Table 3. Delong test to compare the ROC curves of the different model	6

Supplementary Table 4. Performance metrics of the original model and the SMOTE-balanced model evaluated on the independent testing cohort	7

Supplementary Table 5. Logistic Regression coefficients for the PD discriminative model	8

Supplementary Table 6. The specific sequence parameters of the three sequences (3D T1-weighted , ESWAN and ASL imaging)	10

Supplementary Table 7. Comparison of all image features comprising the model in the training and testing cohorts	11

Supplementary Table 8. Comparison of all image features comprising the model in the MSA cohorts	13

Supplementary Table 9. Comparison of all image features comprising the model in the PSP cohorts	15

Supplementary Table 10. Comparison of all image features comprising the model in the ET cohorts	17

Supplementary Table 11. Correlations between the individual predicted scores and clinical measures	19

SUPPLEMENTARY FIGURES	20

Supplementary Fig. 1. The flow chart of the subjects screening	20

Supplementary Fig. 2. The difference analysis of normalized values of the main three image features from SHAP analysis in training cohort and testing cohort....................................................................................................................21

Supplementary Fig. 3. The difference analysis of normalized values of the remaining three image features from SHAP analysis in training cohort and testing cohort	22

Supplementary Fig. 4. The difference analysis of normalized values of the top six image features from SHAP analysis in PSP cohort	23

Supplementary Fig. 5. The difference analysis of normalized values of the top six image features from SHAP analysis in MSA cohort	24

Supplementary Fig. 6. The difference analysis	of normalized values of the top six image features from SHAP analysis in ET cohort	25

SUPPLEMENTARY METHODS	26

A. Sequence parameters	26
B. Quantitative Susceptibility Mapping data processing	26
C. ASL data processing	27
D. The identification and validation of the ASL_PDRP	29

**SUPPLEMENTARY REFERENCES	31**

**SUPPLEMENTARY TABLES**

**Supplementary Table 1. Demographic and clinical information of the MSA and the PSP cohort**

|  | **MSA / PSP cohort** | | | ***P_1_* value** | | ***P_2_* value** |
| --- | --- | --- | --- | --- | --- | --- |
|  | **MSA** | **PSP** | **NC** | |  |  |
| **N** | 18 | 15 | 33 | |  |  |
| **Age (yr)** | 63.39±7.04 | 66.83±10.68 | 62.36±7.10 | | 0.243 | **0.029** |
| **Sex (F/M）** | 10:8 | 6:9 | 22:11 | | 0.433 | 0.082 |
| **MMSE** | 24.67±3.82 | 21.20±6.86 | 29.00 (3.00) | | **0.001** | **<0.001** |
| **Disease duration (yr)** | 2.87±1.77 | 2.78±2.09 | - | |  |  |
| **UPDRS I score** | 1.50 (2.00) | 1.00 (3.00) | - | |  |  |
| **UPDRS II score** | 12.44±5.96 | 15.20±6.61 | - | |  |  |
| **UPDRS III score** | 31.39±13.43 | 37.00±16.70 | - | |  |  |
| **Hoehn-Yahr stage** | 2.75 (2.00-3.00) | 3.00 (3.00-4.00) | - | |  |  |

The one-sample Kolmogorov-Smirnov test is applied to test the normal distribution of data. The normally distributed data is presented as the mean ± SD, while non-normally distributed data is shown as the median (interquartile range). The median (first quartile, third quartile) is shown for the Hoehn-Yahr stage. The independent-samples t test is used for each inter-group comparison of normally distributed data, while the Mann-Whitney U test is used for non-normally distributed data. *P* < 0.050 is considered statistically significant and indicates in bold.

MSA: Multiple System Atrophy; PSP: Progressive Supranuclear Palsy; NC: Normal Controls; MMSE: the Mini-Mental State Examination; UPDRS: United Parkinson’s Disease Rating Scale.

*P_1_*: comparisons between MSA and NC group; *P_2_*: comparisons between PSP and NC group.

**Supplementary Table 2. Demographic and clinical information of the ET cohort**

|  | **ET cohort** | | | ***P* value** | |
| --- | --- | --- | --- | --- | --- |
|  | **ET** | **NC** |  | |  |
| **N** | 80 | 33 |  | |  |
| **Age (yr)** | 59.63±7.77 | 60.62±6.48 | 0.440 | |  |
| **Sex (F/M）** | 46:34 | 22:11 | 0.365 | |  |
| **MMSE** | 28.00 (2.00) | 29.00 (4.00) | 0.170 | |  |
| **Disease duration (yr)** | 10.56 (12.98) | - |  | |  |
| **TETRAS score-2** | 16.25±6.72 | - |  | |  |

The one-sample Kolmogorov-Smirnov test is applied to test the normal distribution of data. The normally distributed data is presented as the mean ± SD, while non-normally distributed data is shown as the median (interquartile range). The independent-samples t test is used for each inter-group comparison of normally distributed data, while the Mann-Whitney U test is used for non-normally distributed data. *P* < 0.050 is considered statistically significant and indicates in bold.

ET: Essential Tremor; NC: Normal Controls; MMSE: the Mini-Mental State Examination; TETRAS: the Essential Tremor Rating Scale.

*P_5_*: comparisons between ET and NC group.

**Supplementary Table 3. Delong test to compare the ROC curves of the different model**

| **Model Comparison** | ***P* values of Delong test (FDR)** | |
| --- | --- | --- |
| **PD-NC_training *vs* MSA-NC** | | 0.617 (0.617) |
| **PD-NC_training *vs* PSP-NC** | | 0.308 (0.411) |
| **PD-NC_training *vs* ET-NC** | | **0.025 (0.100)** |
| **PD-NC_testing *vs* MSA-NC** | | 0.178 (0.284) |
| **PD-NC_testing vs PSP-NC** | | 0.072 (0.143) |
| **PD-NC_testing vs ET-NC** | | 0.450 (0.514) |
| **MSA-NC vs ET-NC** | | **0.041 (0.110)** |
| **PSP-NC vs ET-NC** | | **0.010 (0.083)** |

The Delong test is used to compare the ROC curves of the models obtained from different clinical cohorts, and the *P* values are corrected with false discovery rate.

PD: Parkinson’s Disease; NC: Normal Controls; MSA: Multiple System Atrophy; PSP: Progressive Supranuclear Palsy; ET: Essential Tremor.

PD-NC_training, PD-NC_testing: the PD patients and NC in training and testing cohort; NC referred to the normal controls in testing cohort.

**Supplementary Table 4. Performance metrics of the original model and the SMOTE-balanced model evaluated on the independent testing cohort.**

| **Metrics** | **Original Model** | **SMOTE Model** |
| --- | --- | --- |
| **AUC values (95% CI)** | 0.771 (0.657-0.870) | 0.777 (0.677-0.878) |
| **Sensitivity** | 0.917 | 0.850 |
| **Specificity** | 0.546 | 0.576 |
| **Accuracy** | 0.634 | 0.591 |
| **Precision** | 0.796 | 0.844 |
| **F2 score** | 0.616 | 0.496 |

The original model and SMOTE-balanced model were tested on the same independent testing cohort. A DeLong test comparing the AUCs of the two models yielded a *P* value of **0.324 (Z = - 0.986)**, indicating no statistically significant difference in overall discriminative ability.

SMOTE: the Synthetic Minority Over-sampling Technique; AUC: the Area Under the ROC Curve; CI: Confidence Interval.

**Supplementary Table 5. Logistic Regression coefficients for the PD clinical decision-support model.**

| **Features** | **β weights** | **Std. Error** | **z value** | ***P* value** |
| --- | --- | --- | --- | --- |
| (Intercept) | 1.065 | 0.150 | 7.098 | <0.001 |
| lh_caudalmiddlefrontal | -0.460 | 0.202 | -0.791 | 0.429 |
| lh_inferiorparietal | -0.138 | 0.202 | -0.682 | 0.495 |
| lh_lingual | 0.008 | 0.164 | 0.051 | 0.959 |
| lh_medialorbitofrontal | -0.091 | 0.156 | -0.579 | 0.563 |
| lh_pericalcarine | 0.046 | 0.159 | 0.289 | 0.772 |
| lh_rostralmiddlefrontal | -0.168 | 0.202 | -0.832 | 0.405 |
| rh_caudalanteriorcingulate | -0.061 | 0.151 | -0.403 | 0.687 |
| rh_fusiform | 0.134 | 0.168 | 0.800 | 0.424 |
| rh_inferiortemporal | 0.115 | 0.157 | 0.736 | 0.462 |
| rh_isthmuscingulate | 0.064 | 0.155 | 0.412 | 0.680 |
| rh_parsorbitalis | 0.294 | 0.161 | 1.827 | 0.068 |
| rh_rostralanteriorcingulate | 0.383 | 0.152 | 2.517 | 0.012 |
| rh_supramarginal | 0.704 | 0.189 | 3.729 | <0.001 |
| TIV | 0.098 | 0.154 | 0.634 | 0.526 |
| R_caudate | -0.285 | 0.162 | -1.761 | 0.078 |
| R_pallidum | -0.778 | 0.343 | -2.270 | 0.023 |
| L_SN | 2.511 | 0.301 | 8.345 | <0.001 |
| R_SN | 2.217 | 0.292 | 7.592 | <0.001 |
| ASL_PDRP | 0.719 | 0.165 | 4.351 | <0.001 |

The final logistic regression model can be explicitly expressed as a linear risk score: The individual predicted score = 1.065 - 0.460 × (lh_caudalmiddlefrontal) - 0.138 × (lh_inferiorparietal) + 0.008 × (lh_lingual) - 0.091 × (lh_medialorbitofrontal) + 0.046 × (lh_pericalcarine) - 0.168 × (lh_rostralmiddlefrontal) - 0.061 × (rh_caudalanteriorcingulate) + 0.134 × (rh_fusiform) + 0.115 × (rh_inferiortemporal) + 0.064 × (rh_isthmuscingulate) + 0.294 × (rh_parsorbitalis) + 0.383 × (rh_rostralanteriorcingulate) + 0.704 × (rh_supramarginal) + 0.098 × (TIV) - 0.285 × (R_caudate) - 0.778 × (R_pallidum) + 2.511 × (L_SN) + 2.217 × (R_SN) + 0.719 × (ASL_PDRP).

β weights (Estimate) represents the regression coefficient, i.e., the change in log-odds of PD presence for each one-unit increase in the predictor. Std. Error denotes the standard error of the coefficient estimate, reflecting its precision. The z value is the Wald statistic calculated as β divided by its standard error. The *P* value indicates the probability of observing the given z value, or more extreme, under the null hypothesis that the true coefficient is zero.

TIV: Total Intracranial Volume; SN: Substantia Nigra; ASL_PDRP: the PDRP based on ASL.

**Supplementary Table 6. The specific sequence parameters of the three sequences (3D T1-weighted, ESWAN and ASL imaging).**

| **Parameter Categories** | **Parameter Items** | **Specification / Description** |
| --- | --- | --- |
| **General Information** | Field strength | 3.0T |
|  | Vendor | Discovery MR750, GE Healthcare |
|  | Coil | eight-channel head coil |
|  | Scan coverage | Whole brain |
|  | Patient position | Supine, head-first |
| **3D T1-weighted imaging** | Sequence type | 3D T1w GRE |
|  | TR | 7.336 ms |
|  | TE | 3.036 ms |
|  | TI | 450 ms |
|  | FA | 11° |
|  | FOV | 260 × 260 mm^2^ |
|  | Matrix | 256 × 256 |
|  | slice thickness | 1.2 mm |
|  | axial slices | 196 |
| **ESWAN imaging** | Sequence type | 3D multi-echo GRE |
|  | TR | 33.7 ms |
|  | first echo time / spacing / eighth echo time | 4.556 ms / 3.648 ms / 30.092 ms |
|  | FA | 20° |
|  | FOV | 240 × 240 mm^2^ |
|  | Matrix | 416 × 384 |
|  | slice thickness | 2 mm |
|  | axial slices | 64 |
| **ASL imaging** | Sequence type | background suppressed pseudo-continuous ASL sequence |
|  | TR | 4632 ms |
|  | TE | 10.536 ms |
|  | FA | 111° |
|  | FOV | 240 mm × 240 mm^2^ |
|  | Matrix | 128 × 128 |
|  | slice thickness | 4 mm |
|  | axial slices | 36 |
|  | labeling duration | 1500 ms |
|  | post-labeling delay | 1525 ms |

TR: Repetition Time; TE: Echo Time; FA: Flip Angle; TI: Inversion Time; FOV: Field of View; ESWAN: The Enhanced Susceptibility-weighted Angiography.

**Supplementary Table 7. Comparison of all image features comprising the model in the training and testing cohorts.**

| **Image features** | **Training cohort** | | **Testing cohort** | | ***P* values and t values** | | | |
| --- | --- | --- | --- | --- | --- | --- | --- | --- |
|  | **PD** | **NC** | **PD** | **NC** | ***P_1_* values** | **t_1_ values** | ***P_2_* values** | **t_2_ values** |
| lh_caudalmiddlefrontal | -0.115±0.995 | 0.174±0.962 | -0.159±0.885 | 0.289±1.139 | 0.008 | 2.647 | 0.038 | 2.106 |
| lh_inferiorparietal | 0.001±0.998 | 0.005±0.993 | -0.007±1.026 | 0.012±0.967 | 0.968 | 0.040 | 0.932 | 0.085 |
| lh_lingual | -0.127±1.004 | 0.237±0.926 | -0.260±0.979 | 0.472±0.867 | 0.001 | 3.359 | 0.001 | 3.588 |
| lh_medialorbitofrontal | -0.077±0.980 | 0.148±1.043 | -0.063±1.027 | 0.115±0.954 | 0.043 | 2.033 | 0.414 | 0.820 |
| lh_pericalcarine | -0.089±1.016 | 0.130±0.967 | -0.120±1.034 | 0.219±0.910 | 0.048 | 1.982 | 0.119 | 1.576 |
| lh_rostralmiddlefrontal | -0.119±1.012 | 0.220±0.977 | -0.253±0.835 | 0.459±1.119 | 0.002 | 3.067 | 0.001 | 3.477 |
| rh_caudalanteriorcingulate | -0.089±1.003 | 0.162±0.994 | -0.047±0.920 | 0.086±1.142 | 0.024 | 2.263 | 0.543 | 0.611 |
| rh_fusiform | -0.137±1.010 | 0.207±0.933 | -0.177±0.915 | 0.322±1.080 | 0.002 | 3.149 | 0.020 | 2.362 |
| rh_inferiortemporal | -0.095±1.039 | 0.163±0.926 | -0.149±0.962 | 0.270±1.025 | 0.021 | 2.326 | 0.053 | 1.961 |
| rh_isthmuscingulate | -0.102±1.013 | 0.159±0.961 | 0.019±1.029 | -0.035±0.960 | 0.019 | 2.364 | 0.804 | -0.249 |
| rh_parsorbitalis | -0.056±0.996 | 0.130±0.992 | -0.183±1.025 | 0.332±0.872 | 0.090 | 1.697 | 0.017 | 2.439 |
| rh_rostralanteriorcingulate | 0.041±0.989 | -0.056±1.033 | -0.034±0.959 | 0.062±1.083 | 0.386 | -0.868 | 0.660 | 0.441 |
| rh_supramarginal | -0.001±1.000 | -0.001±0.993 | -0.131±0.990 | 0.239±0.987 | 0.993 | 0.009 | 0.088 | 1.726 |
| TIV | 0.048±0.975 | -0.138±0.956 | 0.092±1.012 | -0.168±0.970 | 0.084 | -1.742 | 0.232 | -1.204 |
| R_caudate | 0.032±0.950 | 0.028±0.831 | 0.020±1.001 | -0.037±1.012 | 0.966 | 0.045 | 0.794 | 0.261 |
| R_pallidum | 0.040±1.039 | -0.036±0.930 | 0.051±0.909 | -0.092±1.157 | 0.597 | 0.679 | 0.616 | 0.659 |
| L_SN | 0.239±1.005 | -0.415±0.782 | 0.229±0.880 | -0.416±1.082 | <0.001 | 6.304 | 0.008 | 3.110 |
| R_SN | 0.222±1.017 | -0.381±0.814 | 0.227±0.834 | -0.412±1.149 | <0.001 | 5.706 | 0.008 | 3.079 |
| ASL_PDRP | 0.138±1.004 | -0.290±0.863 | 0.203±1.066 | -0.368±0.751 | <0.001 | 4.025 | 0.015 | 2.724 |

Values are expressed as mean ± standard deviation or numbers. *P_1_* value and t_1_ value: comparisons between PD and NC group in the training cohort. *P_2_* value and t_2_ value: comparisons between PD and NC group in the testing cohort.

PD: Parkinson’s Disease; NC: Normal Controls; TIV: Total Intracranial Volume; SN: Substantia Nigra; ASL_PDRP: the PDRP based on ASL.

**Supplementary Table 8. Comparison of all image features comprising the model in the MSA cohorts.**

| **Image features** | **MSA cohort** | |  | | | ***P* values and t values** | | | |
| --- | --- | --- | --- | --- | --- | --- | --- | --- | --- |
|  | **MSA** | **NC** | **PD** | ***P_3_* values** | **t_3_ values** | | ***P_4_* values** | **t_4_ values** |  |
| lh_caudalmiddlefrontal | -0.603±0.792 | 0.289±1.139 | -0.159±0.885 | 0.005 | 2.950 | | 0.060 | 1.910 |  |
| lh_inferiorparietal | -0.012±0.996 | 0.012±0.967 | -0.007±1.026 | 0.935 | 0.082 | | 0.985 | 0.018 |  |
| lh_lingual | -0.294±1.046 | 0.472±0.867 | -0.260±0.979 | 0.007 | 2.804 | | 0.898 | 0.129 |  |
| lh_medialorbitofrontal | -0.076±0.937 | 0.115±0.954 | -0.063±1.027 | 0.496 | 0.687 | | 0.963 | 0.047 |  |
| lh_pericalcarine | 0.183±1.068 | 0.219±0.910 | -0.120±1.034 | 0.163 | 1.418 | | 0.822 | 0.225 |  |
| lh_rostralmiddlefrontal | -0.653±0.876 | 0.459±1.119 | -0.253±0.835 | 0.001 | 3.646 | | 0.082 | 1.765 |  |
| rh_caudalanteriorcingulate | 0.017±1.052 | 0.086±1.142 | 0.047±0.920 | 0.834 | 0.210 | | 0.802 | -0.252 |  |
| rh_fusiform | -0.673±0.775 | 0.322±1.080 | -0.177±0.915 | 0.001 | 3.448 | | 0.041 | 2.083 |  |
| rh_inferiortemporal | -0.653±0.762 | 0.270±1.025 | -0.149±0.962 | 0.002 | 3.342 | | 0.045 | 2.036 |  |
| rh_isthmuscingulate | 0.140±0.819 | -0.035±0.960 | 0.019±1.029 | 0.515 | -0.656 | | 0.649 | -0.457 |  |
| rh_parsorbitalis | -0.362±0.931 | 0.204±0.968 | -0.170±0.968 | 0.049 | 2.022 | | 0.108 | 1.625 |  |
| rh_rostralanteriorcingulate | -0.183±0.852 | 0.062±1.083 | -0.034±0.959 | 0.684 | -0.409 | | 0.391 | -0.862 |  |
| rh_supramarginal | -0.342±1.077 | 0.239±0.987 | -0.131±0.991 | 0.057 | 1.946 | | 0.440 | 0.775 |  |
| TIV | 0.018±1.282 | -0.168±0.970 | 0.092±1.012 | 0.563 | -0.582 | | 0.797 | 0.258 |  |
| R_caudate | 0.135±1.067 | -0.074±0.970 | -0.036±0.979 | 0.577 | 0.713 | | 0.931 | 0.583 |  |
| R_pallidum | 0.092±1.248 | -0.050±0.853 | -0.010±0.807 | 0.632 | 0.487 | | 0.931 | 0.167 |  |
| L_SN | 0.575±0.959 | -0.313±0.887 | -0.006±0.835 | 0.007 | 3.031 | | 0.931 | 0.089 |  |
| R_SN | 0.337±1.192 | -0.184±0.842 | -0.123±0.869 | 0.150 | 1.777 | | 0.278 | 2.026 |  |
| ASL_PDRP | 0.563±1.186 | -0.307±0.735 | -0.067±0.968 | 0.007 | 2.968 | | 0.856 | 1.076 |  |

Values are expressed as mean ± standard deviation or numbers. *P_3_* value and t_3_ value: comparisons between MSA and NC group in the MSA cohort. *P_4_* value and t_4_ value: comparisons between MSA and PD group in the MSA cohort.

PD: Parkinson’s Disease; NC: Normal Controls; MSA: Multiple System Atrophy; TIV: Total Intracranial Volume; SN: Substantia Nigra; ASL_PDRP: the PDRP based on ASL.

**Supplementary Table 9. Comparison of all image features comprising the model in the PSP cohorts.**

| **Image features** | **PSP cohort** | |  | | | ***P* values and t values** | | | |
| --- | --- | --- | --- | --- | --- | --- | --- | --- | --- |
|  | **PSP** | **NC** | **PD** | ***P_5_* values** | **t_5_ values** | | ***P_6_* values** | **t_6_ values** |  |
| lh_caudalmiddlefrontal | -0.687±0.892 | 0.289±1.139 | -0.159±0.885 | 0.005 | 2.930 | | 0.043 | 2.064 |  |
| lh_inferiorparietal | -0.364±1.125 | 0.012±0.967 | -0.007±1.026 | 0.241 | 1.187 | | 0.240 | 1.185 |  |
| lh_lingual | -0.522±0.787 | 0.472±0.867 | -0.260±0.979 | <0.001 | 3.787 | | 0.339 | 0.962 |  |
| lh_medialorbitofrontal | -0.670±1.135 | 0.115±0.954 | -0.063±1.027 | 0.016 | 2.490 | | 0.049 | 2.006 |  |
| lh_pericalcarine | -0.476±0.755 | 0.219±0.910 | -0.120±1.034 | 0.013 | 2.579 | | 0.215 | 1.251 |  |
| lh_rostralmiddlefrontal | -0.476±0.755 | 0.219±0.910 | -0.253±0.835 | 0.013 | 2.579 | | 0.019 | 2.400 |  |
| rh_caudalanteriorcingulate | -0.464±0.735 | 0.086±1.142 | -0.047±0.920 | 0.095 | 1.705 | | 0.108 | 1.627 |  |
| rh_fusiform | -0.664±0.889 | 0.322±1.080 | -0.177±0.915 | 0.003 | 3.088 | | 0.068 | 1.853 |  |
| rh_inferiortemporal | -0.357±1.121 | 0.270±1.025 | -0.149±0.962 | 0.062 | 1.909 | | 0.469 | 0.727 |  |
| rh_isthmuscingulate | 0.003±0.689 | -0.035±0.960 | 0.019±1.029 | 0.891 | -0.138 | | 0.954 | 0.058 |  |
| rh_parsorbitalis | -0.752±0.902 | 0.332±0.872 | -0.112±1.008 | <0.001 | 3.951 | | 0.151 | 1.452 |  |
| rh_rostralanteriorcingulate | -0.115±0.746 | 0.062±1.083 | -0.034±0.959 | 0.570 | 0.572 | | 0.763 | 0.303 |  |
| rh_supramarginal | -0.547±1.03 | 0.239±0.987 | -0.131±0.991 | 0.017 | 2.475 | | 0.159 | 1.425 |  |
| TIV | -0.011±1.033 | -0.168±0.970 | 0.092±1.012 | 0.612 | -0.510 | | 0.727 | 0.351 |  |
| R_caudate | 0.472±1.362 | -0.215±0.711 | -0.144±0.780 | 0.030 | 2.207 | | 0.018 | 3.107 |  |
| R_pallidum | 0.677±1.169 | -0.308±0.748 | -0.219±0.721 | 0.001 | 3.160 | | <0.001 | 4.203 |  |
| L_SN | 0.863±1.094 | -0.392±0.661 | -0.209±0.798 | <0.001 | 4.033 | | <0.001 | 3.970 |  |
| R_SN | 0.851±0.978 | -0.387±0.745 | -0.242±0.654 | <0.001 | 3.977 | | <0.001 | 4.776 |  |
| ASL_PDRP | 0.725±1.241 | -0.330±0.660 | -0.108±0.918 | <0.001 | 3.386 | | 0.072 | 1.910 |  |

Values are expressed as mean ± standard deviation or numbers. *P_5_* value and t_5_ value: comparisons between PSP and NC group in the PSP cohort.

*P_6_* value and t_6_ value: comparisons between PSP and PD group in the PSP cohort.

PD: Parkinson’s Disease; NC: Normal Controls; PSP: Progressive Supranuclear Palsy; TIV: Total Intracranial Volume; SN: Substantia Nigra; ASL_PDRP: the PDRP based on ASL.

**Supplementary Table 10. Comparison of all image features comprising the model in the ET cohorts.**

| **Image features** | **ET cohort** | |  | | | ***P* values and t values** | | | |
| --- | --- | --- | --- | --- | --- | --- | --- | --- | --- |
|  | **ET** | **NC** | **PD** | ***P_7_* values** | **t_7_ values** | | ***P_8_* values** | **t_8_ values** |  |
| lh_caudalmiddlefrontal | -0.124±0.948 | 0.289±1.139 | 0.047±1.000 | 0.050 | 1.984 | | 0.634 | -0.478 |  |
| lh_inferiorparietal | 0.101±1.039 | 0.012±0.967 | -0.036±1.179 | 0.993 | 0.009 | | 0.713 | 0.369 |  |
| lh_lingual | 0.027±848 | 0.472±0.867 | 0.020±1.004 | <0.001 | 3.723 | | 0.837 | -0.206 |  |
| lh_medialorbitofrontal | 0.076±0.994 | 0.115±0.954 | -0.101±1.007 | 0.355 | 0.929 | | 0.302 | 1.035 |  |
| lh_pericalcarine | -0.072±1.117 | 0.219±0.910 | 0.096±0.818 | 0.109 | 1.617 | | 0.326 | -0.985 |  |
| lh_rostralmiddlefrontal | 0.089±0.987 | 0.459±1.120 | -0.118±1.013 | 0.003 | 3.032 | | 0.226 | 1.215 |  |
| rh_caudalanteriorcingulate | -0.046±0.870 | 0.086±1.142 | 0.062±1.156 | 0.215 | 1.246 | | 0.527 | -0.634 |  |
| rh_fusiform | -0.005±0.871 | 0.322±1.080 | 0.007±1.158 | 0.006 | 2.797 | | 0.943 | -0.072 |  |
| rh_inferiortemporal | 0.020±0.940 | 0.270±1.025 | -0.026±1.083 | 0.035 | 2.131 | | 0.787 | 0.271 |  |
| rh_isthmuscingulate | -0.095±0.857 | -0.035±0.960 | 0.127±1.159 | 0.894 | 0.133 | | 0.195 | -1.302 |  |
| rh_parsorbitalis | 0.177±1.020 | 0.204±0.968 | -0.237±0.929 | 0.221 | 1.232 | | 0.015 | 2.468 |  |
| rh_rostralanteriorcingulate | -0.035±0.830 | 0.062±1.083 | 0.047±1.196 | 0.286 | 1.071 | | 0.633 | -0.478 |  |
| rh_supramarginal | -0.095±0.951 | 0.239±0.987 | -0.058±1.099 | 0.096 | 1.679 | | 0.552 | 0.596 |  |
| TIV | -0.081±1.115 | -0.168±0.970 | 0.108±0.819 | 0.466 | -0.731 | | 0.272 | -1.103 |  |
| R_caudate | 0.016±0.971 | -0.040±1.081 | 0.021±0.007 | 0.787 | 0.272 | | 0.979 | -0.032 |  |
| R_pallidum | 0.019±0.997 | -0.045±1.022 | 0.028±0.015 | 0.787 | 0.310 | | 0.972 | -0.395 |  |
| L_SN | 0.097±0.914 | -0.237±1.165 | 0.080±0.018 | 0.232 | 1.617 | | 0.095 | 1.998 |  |
| R_SN | 0.097±0.951 | -0.236±1.089 | 0.094±0.015 | 0.232 | 1.610 | | 0.095 | 2.068 |  |
| ASL_PDRP | 0.032±1.026 | -0.077±0.945 | -1.393±1.044 | 0.787 | 0.526 | | 0.017 | 3.046 |  |

Values are expressed as mean ± standard deviation or numbers. *P_7_* value and t_7_ value: comparisons between ET and NC group in the ET cohort.

*P_8_* value and t_8_ value: comparisons between ET and PD group in the ET cohort.

PD: Parkinson’s Disease; NC: Normal Controls; ET: Essential Tremor; TIV: Total Intracranial Volume; SN: Substantia Nigra; ASL_PDRP: the PDRP based on ASL.

**Supplementary Table 11. Correlations between the individual predicted scores and clinical measures.**

| **Clinical measures** | **Correlation coefficients** | ***P* value** |
| --- | --- | --- |
| UPDRS I score | -0.097 | 0.089 |
| UPDRS II score | 0.121 | **0.034** |
| UPDRS III score | 0.138 | **0.015** |
| Purdue Pegboard totally score | -0.209 | **0.010** |
| Purdue Pegboard assembly score | -0.220 | **0.007** |
| Chair test | 0.051 | 0.570 |
| Regular walking speed test | 0.014 | 0.870 |

Chair test: In this study, the patients were required to stand up from a standard armless chair, then sit down repeatedly, for a total of five times. The total time was recorded.

Regular walking speed test: The comfortable walking speed chosen by the patient during a straight-line walking process over a pre-defined distance (usually 5 meters). It is recorded by the total time taken to walk 5 meters. *P* < 0.050 was considered statistically significant and indicated in bold.

UPDRS: Unified Parkinson’s Disease Rating Scale.

**SUPPLEMENTARY FIGURES**

**Supplementary Fig. 1. The flow chart of the subjects screening.** Inclusion and exclusion criteria of PD, NC, MSA, PSP and ET participants in different cohorts.

PD: Parkinson’s Disease; NC: Normal Controls; MSA: Multiple System Atrophy; PSP: Progressive Supranuclear Palsy; ET: Essential Tremor.

**Supplementary Fig. 2. The difference analysis of normalized values of the main three image features from SHAP analysis in training cohort (PD: N = 247, NC: N = 122) and testing cohort (PD: N = 60, NC: N = 33) respectively.** Each data point represented the normalized image feature value for an individual participant. Independent-samples t tests were used to compare group differences in the normalized image features, with adjustments for multiple comparisons using FDR. **Panel A** depicted difference of the ASL_PDRP in training cohort and testing cohort respectively. In training cohort: ASL_PDRP: *P* < 0.001, t = 4.025; in testing cohort: ASL_PDRP: *P* = 0.015, t = 2.724. **Panel B** demonstrated the differences of the R_SN in both cohorts. In training cohort: R_SN: *P* < 0.001, t = 5.706; in testing cohort: R_SN: *P* = 0.008, t = 3.079. **Panel C** showed the differences of the L_SN in both cohorts. In training cohort: L_SN: *P* < 0.001, t = 6.304; in testing cohort: L_SN: *P* = 0.008, t = 3.110. * *P* < 0.05, ** *P* < 0.01, *** *P* < 0.001.

PD: Parkinson’s Disease; NC: Normal Controls; ASL_PDRP: the PD-related spatial covariance pattern based on ASL technology; PD_training, NC_training: the patients with PD and the normal control of training cohort; PD_testing, NC_testing: the patients with PD and the normal control of testing cohort; SN: substantia nigra; FDR: False Discovery Rate. MSA: Multiple System Atrophy; PSP: Progressive Supranuclear Palsy; ET: Essential Tremor. ASL_PDRP: the PDRP based on ASL.

**Supplementary Fig. 3. The difference analysis of normalized values of the remaining three image features (R_pallium, R_caudate, rh_supramarginal) from SHAP analysis in training cohort (PD: N = 247, NC: N = 122) and testing cohort (PD: N = 60, NC: N = 33)**. Each data point represents the normalized feature value for an individual participant. Independent-samples t tests were used to compare group differences in the normalized image features, with adjustments for multiple comparisons using FDR. * *P* < 0.05, ** *P* < 0.01, *** *P* < 0.001.

PD: Parkinson’s Disease; NC: Normal Controls; PD_training, NC_training: the patients with PD and the normal control of training cohort; PD_testing, NC_testing: the patients with PD and the normal control of testing cohort.

**Supplementary Fig. 4. The difference analysis of normalized values of the top six image features (ASL_PDRP, R_SN, L_SN, R_pallium, R_caudate, rh_supramarginal) from SHAP analysis in PSP cohort (PSP: N = 15, PD: N = 60, NC: N = 33)**. In PSP-NC cohort: ASL_PDRP: *P* < 0.001; R_SN: *P* < 0.001; L_SN: *P* < 0.001; R_pallium: *P* = 0.001; R_caudate: *P* = 0.030; rh_supramarginal: *P* = 0.017. In PSP-PD cohort: R_SN: *P* < 0.001; L_SN: *P* < 0.001; R_pallium: *P* < 0.001; R_caudate: *P* = 0.018. Each data point represents the normalized feature value for an individual participant. Independent-samples t tests were used to compare group differences in the normalized image features, with adjustments for multiple comparisons using FDR. * *P* < 0.05, ** *P* < 0.01, *** *P* < 0.001.

PD: Parkinson’s Disease. NC: Normal Controls. PSP: Progressive Supranuclear Palsy. ASL_PDRP: the PDRP based on ASL. PD_testing, NC_testing: the patients with PD and the normal control of testing cohort.

**Supplementary Fig. 5. The difference analysis of normalized values of the top six image features (ASL_PDRP, R_SN, L_SN, R_pallium, R_caudate, rh_supramarginal) from SHAP analysis in MSA cohort (MSA: N = 18, PD: N = 60, NC: N = 33)**. In MSA-NC cohort: ASL_PDRP: *P* = 0.007; L_SN: *P* = 0.007. Each data point represents the normalized feature value for an individual participant. Independent-samples t tests were used to compare group differences in the normalized image features, with adjustments for multiple comparisons using FDR. * *P* < 0.05, ** *P* < 0.01, *** *P* < 0.001.

PD: Parkinson’s Disease. NC: Normal Controls. MSA: Multiple System Atrophy. ASL_PDRP: the PDRP based on ASL. PD_testing, NC_testing: the patients with PD and the normal control of testing cohort.

**Supplementary Fig. 6. The difference analysis of normalized values of the top six image features (ASL_PDRP, R_SN, L_SN, R_pallium, R_caudate, rh_supramarginal) from SHAP analysis in ET cohort (ET: N = 80, PD: N = 60, NC: N = 33)**. In ET-PD cohort: ASL_PDRP: *P* = 0.017. Each data point represents the normalized feature value for an individual participant. Independent-samples t tests were used to compare group differences in the normalized image features, with adjustments for multiple comparisons using FDR. * *P* < 0.05, ** *P* < 0.01, *** *P* < 0.001.

PD: Parkinson’s Disease. NC: Normal Controls. ET: Essential Tremor. ASL_PDRP: the PDRP based on ASL. NC_testing: the patients with PD and the normal control of testing cohort.

**SUPPLEMENTARY METHODS**

A. **Sequence parameters**

High resolution three-dimensional T1-weighted images are acquired with a fast-spoiled gradient-recalled sequence using following parameters: echo time = 3.036 ms; repetition time = 7.336 ms; inversion time = 450 ms; flip angle = 11°; field of view = 260 × 260 mm^2^; matrix = 256 × 256; slice thickness = 1.2 mm; without gap; and 196 axial slices. The enhanced susceptibility-weighted angiography is obtained to generate Quantitative Susceptibility Mapping using gradient recalled echo sequence: repetition time = 33.7 ms; first echo time/spacing/eighth echo time = 4.556 ms/3.648 ms/30.092 ms; flip angle = 20°; field of view = 240 × 240 mm²; matrix = 416 × 384; slice thickness = 2 mm; without gap; 64 continuous axial slices. The ASL images are acquired using a background suppressed pseudo-continuous ASL sequence with the following parameters: repetition time = 4632 ms, echo time = 10.536 ms, flip angle = 111°, slice thickness = 4 mm, without gap, field of view = 240 mm × 240 mm², matrix = 128 × 128, labeling duration = 1500 ms, post-labeling delay = 1525 ms, and 36 axial slices. The specific parameters are listed in the **Supplementary Table 6**.

B. **Quantitative Susceptibility Mapping data processing**

The raw phase images are unwrapped with the Laplacian method^1^ which relied solely on the sine and cosine functions of the phase angle^2^ and the normalized phase is calculated. The normalized background phase is eliminated using the spherical-mean-value filtering method.^3^ Quantitative Susceptibility Mapping images are calculated with the Streaking Artifact Reduction for Quantitative Susceptibility Mapping method.^4^ The mean brain signal of the individual is used as the reference for the susceptibility.

Then the deep learning-based end-to-end tool^5^ is utilized to segment according to the following steps^6^: 1) all the Quantitative Susceptibility Mapping images are co-registered to an age-specific Quantitative Susceptibility Mapping template^7^ using the SyN registration algorithm within the ANTs V2.0 package^8^ and transformation matrices are acquired for each subject; 2) subcortical labels, including caudate nucleus, putamen, globus pallidus, red nucleus, and substantia nigra are created manually in the Quantitative Susceptibility Mapping template space; 3) these labels in the template space are warped to the native space by applying the inverse transformation matrices; and 4) to reduce any voxel misplacement of the surrounding tissue resulting from co-registration bias.^9^ appropriate revisions to the resulting labels are made in the native space. Finally, in the native Quantitative Susceptibility Mapping maps, ten subcortical nuclei from bilateral hemispheres are segmented.

C. **ASL data processing**

The ASL images are automatically converted into CBF maps using Functool software (version 9.4, GE Medical Systems) and preprocessed through Statistical Parametric Mapping (SPM 12, https://www.fil.ion.ucl.ac.uk/spm/software/spm12/) running in Matlab 2018a software (MathWorks, Natick, MA, USA). All CBF maps are visually checked for quality control. The Functool software^10^ performed a series of pre-processing steps during the conversion of ASL images to CBF maps, including CBF calculation, time delay correction, motion correction, interpolation, and spatial alignment. We firstly calculated the CBF for each voxel using the images, followed by post-labeling delay correction to ensure accurate CBF estimation. During the CBF map conversion, the motion parameters of each time point relative to the reference time are calculated by interpolation correction. These parameters are then used to spatially resample the images at each time point, correcting for head motion and ensuring that all images are aligned in the same coordinate system. These steps effectively ensured the high quality and quantitative accuracy of the output CBF images.^11^

ASL and CBF images are normalized by using DARTEL (the exponential lie algebra technique that unified T1 segmentation). Detailed steps are as follows: 1) Tissue segmentation of T1-weighted images: The Segment toolbox in SPM 12 is used to segment the T1-weighted images of each subject into WM, GM, and cerebrospinal fluid. The segmented tissue images are then co-registrated to the standard Montreal Neurological Institute brain space, generating WM and GM templates at group level.^10^ 2) DARTEL registration: individual ASL image is non-linearly registered to the group template. The iterative non-linear registration process of DARTEL enabled accurate alignment of individual brains to the standard space template. 3) ASL and CBF images registration and spatial normalization: The individual ASL images are spatially registered with their CBF images, respectively. Both ASL and CBF images are then spatially normalized and mapped to Montreal Neurological Institute standard space, ensuring spatial consistency between the ASL and CBF images.^11^ We further standardized the CBF map by using z-score. For each individual, the whole-brain mean CBF and standard deviation are used as the reference for normalization to assess the deviation of regional CBF values from the whole-brain level.^12^ The calculated CBF z-scoring helped minimize inter-individual differences in absolute CBF values, reducing the impact of baseline variations and ensuring that CBF measurements across individuals are comparable. Finally, the z-scored CBF maps are spatially smoothed with a Gaussian kernel of 6 × 6 × 6 mm full width at half-maximum,^13^ to improve spatial consistency and reduce the impact of image noise on the analysis.

D. **The identification and validation of the ASL_PDRP**

The preprocessed ASL images are used for the principal component analysis and scaled sub-profile model which is widely employed to identify the PDRP using ScAnVP (Scan Analysis and Visualization Processor, version 7.0w; http://feinsteinneuroscience.org/).^14, 15^ We firstly used the binarized mask of Shen grey matter parcellation^16^ that included gray matter, pons and cerebellar regions which are core regions of classical PDRP. The mask could filter out irrelevant brain areas including ventricles and white matter, guided by a standardized Automatic Anatomical marker template. Then it is smoothed with a full width at half-maximum of 6 mm and multiplied with the CBF maps. The ASL data underwent logarithmic transformation.^17^ double-centering, normalization and adjustment to generate the subject z-scores and principal components which are derived from the covariance matrix via principal component analysis. The first PC, which explains the maximum variance, is always analyzed as the PDRP.^18, 19^ Therefore, the first principal component based on ASL images from our training cohort is regarded as the ASL_PDRP, a major brain imaging feature of PD.

The topographic profile rating assesses the expression of predefined spatial covariance patterns, such as the PD-related spatial covariance pattern.^19^ It provides a subject-specific score reflecting the degree of pattern expression, which can serve as a biomarker for PD. In the topographic profile rating computational procedure, perfusion data from the validation cohort and the reference (ASL_PDRP) are log-transformed and subtracted. The subject score of the validation data relative to the reference is determined by using a multivariate linear regression analysis to predict the topographic profile. The resulting regression coefficient quantifies the difference in topographic profile expression between the the validation data and the reference. In this study, the topographic profile rating is applied quantitatively to assess the expression of ASL_PDRP at the individual level, which served as an image feature in testing and other disease cohorts, respectively.

**SUPPLEMENTARY REFERENCES**

1.	Li W, Avram AV, Wu B, Xiao X, Liu C. Integrated Laplacian-based phase unwrapping and background phase removal for quantitative susceptibility mapping. NMR Biomed. 2014 27(2):219-27.

2.	Li W, Wu B, Liu C. Quantitative susceptibility mapping of human brain reflects spatial variation in tissue composition. Neuroimage. 2011 55(4):1645-56.

3.	Wu B, Li W, Guidon A, Liu C. Whole brain susceptibility mapping using compressed sensing. Magn Reson Med. 2012 67(1):137-47.

4.	Wei H, Zhang Y, Gibbs E, Chen NK, Wang N, Liu C. Joint 2D and 3D phase processing for quantitative susceptibility mapping: application to 2D echo-planar imaging. NMR Biomed. 2017 30(4).

5.	Guan Y, Guan X, Xu J, Wei H, Xu X, Zhang Y. DeepQSMSeg: A Deep Learning-based Sub-cortical Nucleus Segmentation Tool for Quantitative Susceptibility Mapping. Annu Int Conf IEEE Eng Med Biol Soc. 2021:3676-9.

6.	Guan X, Guo T, Zhou C, Wu J, Gao T, Bai X, et al. Asymmetrical nigral iron accumulation in Parkinson's disease with motor asymmetry: an explorative, longitudinal and test-retest study. Aging (Albany NY). 2020 12(18):18622-34.

7.	Zhang Y, Wei H, Cronin MJ, He N, Yan F, Liu C. Longitudinal atlas for normative human brain development and aging over the lifespan using quantitative susceptibility mapping. Neuroimage. 2018 171:176-89.

8.	Avants BB, Epstein CL, Grossman M, Gee JC. Symmetric diffeomorphic image registration with cross-correlation: evaluating automated labeling of elderly and neurodegenerative brain. Med Image Anal. 2008 12(1):26-41.

9.	Guan X, Guo T, Zhou C, Wu J, Zeng Q, Li K, et al. Altered brain iron depositions from aging to Parkinson's disease and Alzheimer's disease: A quantitative susceptibility mapping study. Neuroimage. 2022 264:119683.

10. Okonkwo OC, Xu G, Oh JM, et al. Cerebral blood flow is diminished in asymptomatic middle-aged adults with maternal history of Alzheimer's disease. Cereb Cortex. 2014 24(4):978-988.

11.	Cao Z, Wu C, Hong H, Huang P, Zhou C, Guan X, et al. Predictability of inter-regional cerebral perfusion similarity on dopamine responsiveness and the moderation role of cognition in PD patients. Neuroimage. 2023 279:120305.

12. Wu C, Wu H, Zhou C, et al. Neurovascular coupling alteration in drug-naïve Parkinson's disease: The underlying molecular mechanisms and levodopa's restoration effects. Neurobiol Dis. 2024 191:106406.

13.	Shang S, Ye J, Wu J, Zhang H, Dou W, Krishnan Muthaiah VP, et al. Early disturbance of dynamic synchronization and neurovascular coupling in cognitively normal Parkinson's disease. J Cereb Blood Flow Metab. 2022 271678X221098503.

14.	Eidelberg D. Metabolic brain networks in neurodegenerative disorders: a functional imaging approach. Trends in Neurosciences. 2009 32(10):548-57.

15.	Spetsieris PG, Eidelberg D. Scaled subprofile modeling of resting state imaging data in Parkinson's disease: methodological issues. Neuroimage. 2011 54(4):2899-914.

16.	Barron DS, Salehi M, Browning M, Harmer CJ, Constable RT, Duff E. Exploring the prediction of emotional valence and pharmacologic effect across fMRI studies of antidepressants. Neuroimage Clin. 2018 20:407-14.

17.	Rane S, Koh N, Oakley J, Caso C, Zabetian CP, Cholerton B, et al. Arterial spin labeling detects perfusion patterns related to motor symptoms in Parkinson's disease. Parkinsonism Relat Disord. 2020 76:21-8.

18.	Wu P, Wang J, Peng S, Ma Y, Zhang H, Guan Y, et al. Metabolic brain network in the Chinese patients with Parkinson's disease based on 18F-FDG PET imaging. Parkinsonism Relat Disord. 2013 19(6):622-7.

19.	Tomše P, Jensterle L, Grmek M, Zaletel K, Pirtošek Z, Dhawan V, et al. Abnormal metabolic brain network associated with Parkinson's disease: replication on a new European sample. Neuroradiology. 2017 59(5):507-15.
